# Lower IgG somatic hypermutation rates during acute dengue virus infection is compatible with a germinal center-independent B cell response

**DOI:** 10.1186/s13073-016-0276-1

**Published:** 2016-02-25

**Authors:** Elizabeth Ernestina Godoy-Lozano, Juan Téllez-Sosa, Gilberto Sánchez-González, Hugo Sámano-Sánchez, Andrés Aguilar-Salgado, Aarón Salinas-Rodríguez, Bernardo Cortina-Ceballos, Héctor Vivanco-Cid, Karina Hernández-Flores, Jennifer M. Pfaff, Kristen M. Kahle, Benjamin J. Doranz, Rosa Elena Gómez-Barreto, Humberto Valdovinos-Torres, Irma López-Martínez, Mario H. Rodriguez, Jesús Martínez-Barnetche

**Affiliations:** Centro de Investigación Sobre Enfermedades Infecciosas, Instituto Nacional de Salud Pública, Cuernavaca, Morelos México; Centro de Investigación en Evaluación y Encuestas, Instituto Nacional de Salud Pública, Cuernavaca, Morelos México; Instituto de Investigaciones Médico-Biológicas, Universidad Veracruzana, Veracruz, Veracruz México; Integral Molecular Inc., Philadelphia, PA USA; Instituto de Diagnóstico y Referencia Epidemiológicos, México, DF México

## Abstract

**Background:**

The study of human B cell response to dengue virus (DENV) infection is critical to understand serotype-specific protection and the cross-reactive sub-neutralizing response. Whereas the first is beneficial and thus represents the ultimate goal of vaccination, the latter has been implicated in the development of severe disease, which occurs in a small, albeit significant, fraction of secondary DENV infections. Both primary and secondary infections are associated with the production of poly-reactive and cross-reactive IgG antibodies.

**Methods:**

To gain insight into the effect of DENV infection on the B cell repertoire, we used VH region high-throughput cDNA sequencing of the peripheral blood IgG B cell compartment of 19 individuals during the acute phase of infection. For 11 individuals, a second sample obtained 6 months later was analyzed for comparison. Probabilities of sequencing antibody secreting cells or memory B cells were estimated using second-order Monte Carlo simulation.

**Results:**

We found that in acute disease there is an increase in IgG B cell diversity and changes in the relative use of segments *IGHV1-2*, *IGHV1-18*, and *IGHV1-69*. Somewhat unexpectedly, an overall low proportion of somatic hypermutated antibody genes was observed during the acute phase plasmablasts, particularly in secondary infections and those cases with more severe disease.

**Conclusions:**

Our data are consistent with an innate-like antiviral recognition system mediated by B cells using defined germ-line coded B cell receptors, which could provide a rapid germinal center-independent antibody response during the early phase of infection. A model describing concurrent T-dependent and T-independent B cell responses in the context of DENV infection is proposed, which incorporates the selection of B cells using hypomutated IGHV segments and their potential role in poly/cross-reactivity. Its formal demonstration could lead to a definition of its potential implication in antibody-dependent enhancement, and may contribute to rational vaccine development efforts.

**Electronic supplementary material:**

The online version of this article (doi:10.1186/s13073-016-0276-1) contains supplementary material, which is available to authorized users.

## Background

Dengue, the most prevalent mosquito borne-viral disease in humans, is caused by four closely related serotypes of dengue virus (DENV) of the Flaviviridae family. The infection can be asymptomatic, produce a mild self-limited febrile disease lasting up to 10 days, or result in a severe disease with hemostatic and hemodynamic dysfunction [1, 2]. According to recent estimates, 390 million dengue infections occur each year, of which only 96 million reach the public health surveillance system [3].

DENV primarily infects dendritic cells and monocytes [4–6]. During the early stages of infection, a rapid, strong innate immune response is elicited, resulting in a systemic inflammatory immune response that explains the majority of symptoms of uncomplicated illness. Shortly after, an adaptive immune response manifests as the production of low-affinity IgM anti-DENV antibodies, and, later, high-affinity anti-DENV IgG-neutralizing antibodies that confer long-term protection to the infecting serotype (homotypic protection). However, primary infections are also associated with the production of low-affinity, non-neutralizing or sub-neutralizing cross-reactive antibodies against multiple DENV-serotypes that only confer partial short-term protection [7].

An almost paradigmatic feature of dengue infection is the epidemiological observation that severe illness is associated with a previous infection with a different DENV serotype [8]. Non-neutralizing cross-reactive antibodies induced during primary infections have been implicated in potentiating viral replication, systemic immunopathology, and severe disease by FcγR-mediated antibody-dependent enhancement (ADE), offering a plausible explanation of the increased disease severity after multiple reinfections [9].

Antibody production is the result of B cell clonal selection, expansion, and differentiation into antibody-secreting plasma cells. In the case of protein antigens, antigen-specific B cells are clonally selected and form germinal centers (GC) depending on T cell help. Within the GC, selected B cells undergo somatic hypermutation (SHM) and class switch recombination (CSR) [10, 11], which are two mechanistically coupled processes resulting in high affinity IgG-switched memory B (mB) cells and antibody-secreting plasma cells [12]. However, it has become increasingly clear that B cell memory is heterogeneous in terms of their origin and function [13]. In the context of DENV infection, the generation of long-lived anti-DENV IgG^+^ memory cells selected by a primary infection has been implicated in the predominance of neutralizing antibodies against the primary infecting serotype over neutralizing antibodies specific for a different serotype causing a secondary infection [14]. Presumably, this so-called original antigenic sin could also favor ADE by outcompeting secondary serotype anti-DENV high-affinity B cell clones. Consistently, poly-reactive and serotype cross-reactive IgGs are present in high titers in early primary and secondary DENV infections [15–19]. Thus, understanding B cell responses to DENV infection as well as antibody-mediated immune protection and enhancement requires the integrative analysis of the clonal selection process within the actual conceptual framework of pathway heterogeneity of effector B cell differentiation [13].

Given the central role of antibodies and their affinity in the protection against DENV and their implication in severe disease, we hypothesized that the B cell clonal selection process and diversification may be different between primary and secondary dengue infections, and between DENV infections without clinical warning signs (DWS−) and dengue infections with clinical warning signs (DWS+). To identify these differences, we used high-throughput sequencing (HTS) of peripheral blood IgG antibody repertoires derived from cDNA (reviewed in [20, 21]) to compare clonal diversity, IGHV usage, and SHM rates between patients with acute DWS− and DWS+ and their corresponding post-convalescent blood samples (6 months after).

We found differential changes in *IGHV1-2*, *IGHV1-18*, and *IGHV1-69* clonal usage frequency and transcription. Paradoxically, we observed overall lower SHM rates during acute illness, particularly in DWS+ and in lymphocytes using *IGHV1-2*, suggesting their implication in cross-reactive low-affinity IgG antibodies. Our results also suggest that in humans infected with DENV, in addition to a classical GC pathway, an alternative GC-independent effector B cell differentiation pathway may take place, in which CSR to IgG is decoupled from SHM.

## Methods

### Ethics statement

This study was conducted according to the principles expressed in the Declaration of Helsinki. The study was approved by the Research, Ethics and Biosafety Committees of the Instituto Nacional de Salud Pública (CI:1023/1100), Universidad Veracruzana, Integral Molecular and Instituto de Diagnóstico y Referencia Epidemiológicos (InDRE). Written informed consent was obtained from all participants.

### Patients, donors, and samples

During dengue season 2010 and 2011, 19 adult patients with clinical and laboratory confirmed DENV infection living in Veracruz, a DENV endemic zone in Mexico [22], were enrolled after providing written informed consent. Patients were classified as DWS+ if they required hospitalization, had hematocrit > 40, a platelet count < 100 × 10^3^, and at least one of the following signs: abdominal pain or tenderness, persistent vomiting, clinical fluid accumulation, mucosal bleeding, lethargy, restlessness, or liver enlargement (>2 cm). Patients donated two peripheral blood samples to provide total RNA and serum: one during the febrile stage (acute sample) and the other 6 months after recovery (post-convalescent sample), coinciding with the low transmission season to minimize the possibility of asymptomatic reinfection. For some data analyses, an additional control group of 10 healthy volunteers enrolled in an influenza vaccination study [23], was included. Peripheral blood was collected to obtain serum and total RNA. For RNA isolation, 2.5 ml of peripheral blood in PaxGene RNA tubes (QIAGEN GmbH, Hilden, Germany) was used according to manufacturer’s instructions and stored at −70 °C until use.

### DENV serological diagnosis of primary and secondary cases

All sera were tested with reference anti-NS1 enzyme-linked immunosorbent assay (ELISA) with the Platelia Dengue NS1 Ag Test (Bio-Rad, Marnes-la-Coquette, France) and Dengue IgM (Panbio®, Sinnamon Park, QLD, Australia). To discriminate between primary and secondary infections, we used the IgG Capture ELISA (Panbio®). Primary cases were defined as a positive reverse-transcriptase polymerase chain reaction (RT-PCR), NS1, and/or anti-IgM ELISA and negative anti-IgG ELISA. Secondary cases were defined as positive anti-IgG ELISA and NS1/or RT-PCR, regardless of the results of the anti-IgM ELISA.

### Dengue virus genotyping

Viral RNA was isolated from the sera during the acute febrile phase (QIAamp Viral RNA mini kit, Qiagen) and used to determinate DENV serotype by quantitative RT-PCR according to the protocol of InDRE, Mexico [24], and the Official Mexican Norm: NOM-032-SSA-2002 [25].

### DENV reporter virus particles neutralization assay

DENV reporter virus particles (RVPs) for the four serotypes [26] were pre-incubated with an equal volume of all serially diluted acute sera (1:10 to 1:10,240; all dilutions were pre-incubated with RVPs) in complete Dulbecco’s Modified Eagle Medium for 1 h at room temperature with slow agitation. Following incubation, BHK DC-SIGN cells were added to each well at a density of 30,000 cells per well, followed by incubation at 37 °C in 5 % CO_2_ for 72 h. Cells were subsequently lysed and analyzed for luciferase luminescent reporter expression. The percent infection for each concentration of serum was calculated using Prism software 5.0 and raw data expressed as percent infection versus log_10_ of the reciprocal serum dilution. A sigmoidal dose–response curve with a variable slope was applied to determine the titer of antibody that achieved a 50 % reduction in infection (50 % neutralization titer, NT_50_). Maximum infection was determined using a no serum control. An NT_50_ ≥ 1:50 was defined as a positive neutralization test.

### VH libraries and high throughput cDNA sequencing

The RNA concentration and integrity was analyzed through capillary electrophoresis in an Agilent 2100 BioAnalyzer, with the RNA 6000 Pico kit. cDNA was generated for the VH region of IgG through 5′ rapid amplification of cDNA ends (RACE)-PCR, using a protocol modified from the SMART RACE cDNA Amplification Kit (Clontech Laboratories, Inc.). The forward primer (FpAmpTA) was a modification from the UPM primer to which we added to the 5′ end an A adapter from the platform GS FLX Titanium 454-Roche. The reverse primer TBIgGHu [5′-(454adaptorB)CTA TGC GCC TTG CCA GCC CGC (454key) TCAG(IGHG)ACC GAT GGG CCC TTG GTG-3′] primes in Exon I of the *IGHG* genes and has the B adapter for the 454-Roche sequencing [27]. We analyzed the 500–600 bp 5′RACE-PCR products with 1.5 % agarose gel electrophoresis and purified them with a MiniElute PCR purification kit (Qiagen). Their concentration and integrity was analyzed through capillary electrophoresis in a 2100 BioAnalyzer using the High Sensitivity DNA 2100 LabChip (Agilent Technologies).

We used 100 ng of each library for the emulsion PCR (GS emPCR Kit, 454-Roche). HTS was performed using Genome Sequencer FLX Titanium System 454-Roche with the GS LR70 Sequencing Kit according to the manufacturer’s instructions. This platform generates an average read length of 450–500 bp. We performed the sequencing with the B adapter (3′ → 5′) so that the complementarity determining region heavy 3 (CDRH3) region was proximal and the 5′ UTR was the sequencing primer, allowing higher sequencing quality in the majority of the *IGHV* coding region. Raw sequencing files are available in NCBI-SRA: BioProject ID: PRJNA302665; accession number: SAMN04277236-65.

### Bioinformatics analysis

#### Estimation of probabilities of sampling either IgG^+^ antibody-secreting cells or memory B cells

To overcome the limitation of working with unsorted IgG^+^ B cell subpopulations [antibody-secreting cells (ASC) or mB], we designed a computational protocol consisting of a second-order Monte Carlo simulation to estimate the probability of picking a progressive number of clonally related sequencing reads belonging to either population for each cell sampled, during the acute disease and post-convalescence [28]. The model accounts for individual variation over a gradient of relative proportions of ASC and mB cells, as well as cellular variation in the relative Ig transcription levels in both subpopulations. Briefly, the protocol calculates the probability of sampling Ig transcripts from either subpopulation by random sampling distributions corresponding to the relative amount of either subpopulation in a blood sample, as well as the relative amount of Ig transcripts per cell. The process is calculated in 500 individuals, for a given mB cell to ASC ratio that begins with 1 % of ASCs in post-convalescent individuals and ends with 1 % of mB cells (Additional file 1). In the simulation, an average of 1000 IgG^+^ B cells having a normal distribution and 5 % variance were randomly sampled. The Ig expression in mB cells has a normal distribution with a mean of 100 arbitrary units (au) and 5 % variance, and the Ig expression in ASCs follows a gamma distribution with central value of 1200 au (12-fold increase relative to an mB cell), a minimal value of 300, and a maximal value of 10,000 au [29] (Additional file 1).

### Pre-processing and repertoire reconstruction

We have developed a software (pipeline) named *ImmunediveRsity* for the analysis of the repertoire sequencing (Rep-Seq) data [30]. *ImmunediveRsity* is written in R language [31] and automates Ig sequencing analysis from pre-processing, error correction and quality filtering, V(D)J segment assignment, CDRH3-based sequence clustering for heavy chain clonotypes, and their further clustering into heavy chain lineages as a result of clonotype diversification by SHM (referred hereafter as clonotypes and lineages, respectively. Additional file 2). Raw sequences with an average ≥ Q28 value and reads ≥250 bp passed the quality filter. In order to exclude non-VH sequences, *ImmunediveRsity* assigns IGHV and IGHJ segment use to each read using IgBLAST [32]. A clonotype is composed by reads that share the same V and J segment and their CDRH3 has the same length and is 97 % identical [30]. To discard a possible effect of a CDRH3 clustering threshold on SHM, repertoire data are also reconstructed at a 92 % identity threshold. Reads belonging to a clonotype are further clustered along the whole coding region, excluding the signal peptide, so that the lineage is the consensus of reads sharing 99.5 % identity (Additional file 2). For the analysis of IGHV usage, collapsing sequences according to a common clonal origin and to a particular lineage allows the frequency to be expressed according to total clonotypes or lineages, regardless of Ig transcription levels. Thus, a given clonotype composed of 80 % of the sequencing reads has an equal clonotype frequency to that of a clonotype composed of 0.1 % of the sequenced reads. The same applies for lineages. For *ImmunediveRsity*, a lineage is an approximation of a single B cell, although it is possible to underestimate the true B cell numbers (for example, when two B cells from the same clonotype are identical or the proportion of SHM is below the clustering threshold of 99.5 % identity). *ImmunediveRsity* output files for each sequenced library can be found at http://201.131.57.23:8080/dengue-project-2015/.

### Estimation of sample B cell diversity

*ImmunediveRsity* calculates clonotype and lineage entropy values (Shannon Index) [33, 34] and performs a rarefaction analysis [35] as indirect measures of lymphocyte diversity. The number of reads per clonotype and lineage obtained for each sample (acute or post-convalescent phase) was used to calculate the Shannon Index. Rarefaction curves were calculated with the number of clonotypes in growing subsamples of 1000 reads.

### IGHV segment overrepresentation analysis

We used three approaches to identify overrepresented IGHV segments during acute DENV infection. The first approach aimed to reflect IGHV use based on the estimation of IGHV segment relative transcription levels, regardless of clonotype and lineage composition, and was calculated based on the proportion of reads for each IGHV family and segments normalized to the total number of reads per library (raw IGHV usage). When both acute and post-convalescent samples were available, the proportion of reads for each segment during the acute (A) phase was subtracted from its corresponding value during the post-convalescent (Pc) phase (ΔA–Pc). The second and third approaches aimed to estimate IGHV usage per clonotype or lineage, respectively, where the number of clonotypes or lineages using a particular IGHV segment was expressed as the proportion of all clonotypes or lineages in the corresponding library using a particular IGHV segment. Similar to the first approach, changes of IGHV usage are expressed as the difference of the acute phase frequency minus its corresponding post-convalescent frequency (ΔA − Pc). Statistical evaluation was done with a two-way analysis of variance (ANOVA) with Bonferroni correction for multiple testing using Graph Pad Prism v5.0. Differences were considered statistically significant if *p* < 0.05.

### VH mutation analysis

The numbers of non-synonymous and synonymous mutations were obtained with IMGT/HighVQuest [36] for each lineage consensus. To compare the proportion of mutations, only productive lineages were used for random sub-sampling (1280 lineages per library, which corresponded to the library with the least number of lineages). The proportion of mutations (pM-VH), proportion of non-synonymous mutations, and the proportion of synonymous mutations were calculated as the percentage of total mutations in the VH region, excluding the CDRH3, divided by its length. To avoid non-independence effects from lineages derived from large clonotypes, SHM was also calculated in the largest lineage per clonotype from 250 randomly sampled clonotypes. To identify differences in the proportion of mutations per IGHV segment, the mean global mutation proportion was subtracted from each individual IGHV mean mutation proportion. The difference was used for unsupervised hierarchical clustering according to IGHV segment using an uncentered correlation metric for clustering with CLUSTER 3.0 [37]. We performed multivariate comparisons among control, DWS− A, DWS+ A, DWS− Pc, and DWS+ Pc consensus of lineages based on different metrics including the mean of the proportion of mutations (non-synonymous and synonymous), the mean frequency of lineages, and CDRH3 length. A multilevel principal component analysis [38] was applied in a sample of 1280 randomly chosen lineages for each individual, and graphically visualized using biplot graph, which is a graphical representation of principal component 1 (PC1) versus principal component 2 (PC2), which are selected by the proportion of explained variance (that is, accounting for as much of the variability in the data as possible). This analysis was conducted using R software [39]. Non-parametric analyses (Kruskal–Wallis test) with Dunn’s correction for multiple testing were performed for comparisons among the different groups with R software [40]. Differences were considered statistically significant if *p* < 0.05.

### CDRH3 convergence analysis

CDRH3 convergent signatures have been described in acute DENV infection [41]. We used two approaches to identify CDRH3 convergent signatures: the first was based on searching for the previously described signatures in our VH clonotype databases in acute infection and post-convalescence. The second was based on de novo identification of shared CDRH3 within our datasets. For both approaches, the R function *Find_CDR3* of *ImmunediveRsity* was used [30].

## Results

### Donors, samples, demographic data, and sequencing metrics

**Fig. 1:**
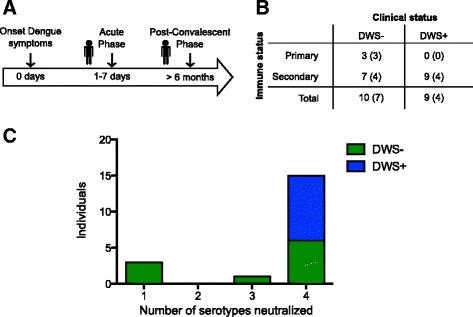
Clinical specimen sampling design and DENV cross-neutralization. **a** Graphical representation of peripheral blood samples in relation to the onset of symptoms. **b** Clinical and immune status of patients included in the study. The number of patients in each category during the acute phase is shown. Number of post-convalescent samples is shown in parentheses. **c** Cross-reactivity of acute DENV infection sera to four serotypes using the DENV reporter virus particle neutralization test. Each bar represents the number of individuals having NT_50_ > 1:50 to *x* number of serotypes. *DWS−* dengue without warning signs; *DWS+* dengue with warning signs

In order to characterize the impact of acute DENV infection in the human B cell repertoire in terms of clinical status (DWS− and DWS+) and immune status (primary and secondary infection), we sampled peripheral blood from 19 patients with laboratory-confirmed DENV infection during their febrile stage (DWS− A, *n* = 10; DWS+ A, *n* = 9). No differences in the number of days after onset of symptoms were found regarding clinical (DWS− or DWS+) (Additional file 3, Table 1) or immune status. As a reference, a second sample was obtained from some individuals 6 months after the first sample (post-convalescence) (DWS− Pc = 7, DWS+ Pc = 4) (Fig. 1a, b). Socio-demographic and clinical data are summarized in Table 1. Of the 19 patients, only three had a primary infection (15.8 %) during the acute stage and the rest had secondary infections (84.2 %). All primary cases were classified as DWS−. The predominant infecting serotype was DENV2 (10/19; 52.6 %), followed by DENV1 (7/19; 36.8 %). We could not determine the serotype in four patients (21.0 %) (Table 1). Sera from 15 individuals (78.9 %) presented high titers of cross-neutralizing antibodies to the four DENV serotypes, as measured by DENV reporter particle neutralization assay [26]; one individual showed cross-reactive titers to three DENV serotypes; and, as expected, the three patients with primary infections showed homotypic neutralization (Fig. 1c, Additional file 4). Owing to the high cross-reactivity among DENV serotypes, it was not possible to identify which was the primary infecting serotype in secondary cases.

**Table 1:** Demographic data and clinical parameters

		DWS− A	DWS− Pc	DWS+ A	DWS+ Pc
Number of individuals, *n*		10	7	9	4
Male, n (%)		3 (30 %)	2 (28.6 %)	4 (44.4 %)	1 (25 %)
Age in years, median (range)		33 (18–50)	27 (18–50)	30 (16–47)	18 (16–46)
Days after symptom onset, median (range)		4 (1–8)	294.5 (133–323)	6 (3–9)	268 (128–309)
Type of infection, n (%)	Primary	3 (30 %)	3 (42.9 %)	0 (0 %)	0 (0 %)
	Secondary	7 (70 %)	4 (57.1 %)	9 (100 %)	4 (100 %)
Serotype, n (%)	*DENV1*	2 (20 %)	NA	3 (33.3 %)	NA
	*DENV2*	5 (50 %)	NA	5 (55.6 %)	NA
	Unknown	3 (30 %)	NA	1 (11.1 %)	NA
Hemoglobin, mean (SD)		14.2 (1.5)	UD	13.55 (2.0)	UD
Platelets, mean (SD)		125.5 (78.0)	UD	68.8 (34.7)	UD

*A* acute, *DWS−* dengue without warning signs; *DWS+* dengue with warning signs; *NA* not applicable, *Pc* post-convalescence, *SD* standard deviation, *UD* undetermined. Percentages (%) are calculated based on n per column

**Fig. 2:**
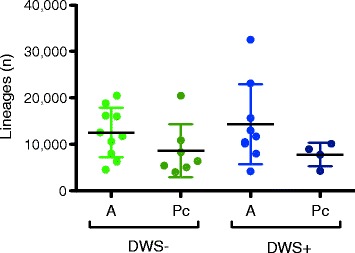
Number of lineages by clinical status. Absolute number of heavy chain lineages (*ImmunediveRsity* output) in acute DENV infection and the post-convalescent period according to clinical status (one-way ANOVA, Bonferroni correction for multiple testing). *A* acute, *DWS−* dengue without warning signs; *DWS+* dengue with warning signs; *NA* not applicable, *Pc* post-convalescence

Thirty VH region IgG^+^ cDNA libraries of peripheral blood B cells were generated using a generic IgHG CH1-coding exon-specific antisense oligonucleotide for 5′RACE-PCR amplification. A total of 2,364,822 raw and 2,044,447 pass-filter 454-Roche sequences were generated [27]. Pass filter reads were used as the input for *ImmunediveRsity* [30], which reconstructed 385,206 heavy chain lineages derived from 146,565 heavy chain clonotypes. The average number of lineages and clonotypes per patient was 11,553 (±6587) and 4420 (±2961), respectively (Table 2, Additional file 5). During acute dengue infection, there is a massive mobilization of plasmablasts to peripheral blood [42], and we identified a higher number IgG lineages during acute dengue infection (Fig. 2). Rarefaction analyses for clonotypes and entropy measurements were consistent with a higher number of IgG B cells during acute infection (Additional file 6). Given that the source of the sequenced material was RNA, these results imply that the sequenced lineages either had higher IgG expression (i.e., plasma cells and plasmablasts) or were clonally expanded.

**Table 2:** Sequencing summary

	DWS− A		DWS− Pc		DWS+ A		DWS+ Pc	
	Total	Media ± SD	Total	Media ± SD	Total	Media ± SD	Total	Media ± SD
Raw sequences	731,977	73,197 ± 25,500	460,685	65,812 ± 23,151	594,743	66,082 ± 27,433	260,066	65,016 ± 47,798
Pass filters sequences	659,621	65,962 ± 23,636	395,217	56,459 ± 22,634	499,483	55,498 ± 25,926	227,017	56,754 ± 44,401
Heavy chain clonotypes (unique)	49,616	4961 ± 2590	19,260	2751 ± 858	53,573	5952 ± 4027	10,144	2536 ± 806
Heavy chains lineages (unique)	125,631	12,563 ± 5334	60,708	8672 ± 5687	129,040	14,337 ± 8620	31,213	7803 ± 2534

*A* acute, *DWS−* dengue without warning signs; *DWS+* dengue with warning signs; *NA* not applicable, *Pc* post-convalescence, *SD* standard deviation; media and SD was calculated by the number of individuals in each group

### Estimation of the cellular composition and origin of sequencing reads

**Fig. 3:**
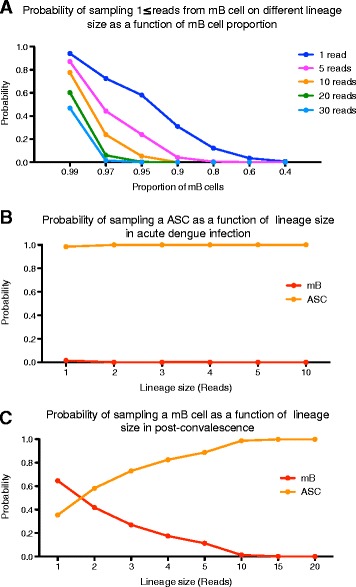
Probability of sampling memory B (mB) cells and antibody-secreting cells (ASCs) in the Rep-Seq output by Monte Carlo simulation. The probability of sampling an mB cell of a given size (number of reads per lineage) was calculated as described in Additional file 1. **a** Probability of sampling an mB cell of a given lineage size (read number) as a function of the proportion of mB cells in the sample (*x* axis). **b** Simulation of a dengue infection where plasmablast mobilization increases to 56 % of IgG^+^ B cells (or ~5.6 % of CD19+), with a concomitant reduction in the relative proportion of mB cells to 44 %. **c** Simulation of post-convalescence where the proportion of plasmablasts returns to basal levels (4.3 % of IgG^+^) and mBs dominate the sample. In these conditions, only very large lineages (≥10 reads) are expected to derive from ASCs

Results from the Monte Carlo simulation showed that the probability of sampling an mB cell lineage larger than 5 reads drops rapidly when the mB to ASC ratio drops below 9:1 (Fig. 3a, Additional file 1). Because the actual number of ASC and mB cells in our samples is unknown, we estimated the probability of sampling an IgG^+^ mB based on the previously described average IgG^+^ plasmablast count (56 %) during acute dengue infection (approximately 5.6 % of CD19+ B cells). Using these parameters, the probability of sampling a single read from an mB cell lineage was 0.015 and decreased for larger clonotypes (Fig. 3b. Additional file 1). However, even with a modest plasmablast increase to a proportion of 10 % (mB to ASC ratio of 9:1, or 1 % of CD19+ B cells), the probability of sampling an mB cell lineage larger than 5 reads was <0.04 (Fig. 3a, Additional file 1).

This scenario changes substantially in the post-convalescent phase, where the predominant IgG-expressing B cells are mB (average 95.8 %) [43, 44]. Under these conditions, sampling one read derived from an mB cell is common (*p* = 0.64) and its probability decreases below 0.012 until a lineage size threshold of 10 reads or higher is reached (Fig. 3c). The results of Monte Carlo simulation indicated that in patients with acute dengue, the majority of sequencing reads and the resulting lineages derived from ASCs. In contrast, during post-convalescence, lineages above 10 reads mainly derived from ASCs. However, we cannot exclude the possibility that in patients sampled at early-symptom onset, the proportion of ASCs may be similar to those sampled during the post-convalescent phase.

### Differential usage of IGHV families and segments in acute phases

**Fig. 4:**
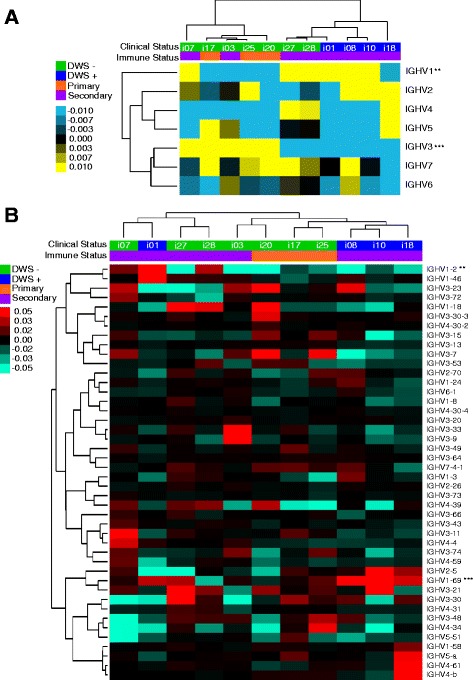
Preferential IGHV usage during acute DENV infection. **a** Heat map of hierarchical clustering of the difference between acute minus post-convalescent lineage frequency (ΔA − Pc) per IGHV family. Overrepresentation of IGHV family usage during acute phase is indicated in *yellow* tones, whereas *blue* tones indicate underrepresentation. *Upper rows* classify patients according to clinical status (DWS− *green* and DWS+ *blue*) and immune status (primary *orange* and secondary *purple*) (two-way ANOVA, Bonferroni correction for multiple testing, *p < 0.05). **b** Difference between acute minus post-convalescent (ΔA − Pc) according to IGHV segment, clinical status, and immune status. *IGHV1-2* and *IGHV1-69* are overrepresented in acute DWS+ (*columns*, DWS− *green* and DWS+ *blue*) and immune status (primary *orange* and secondary *purple*). A higher frequency of lineages using *IGHV1-18* and *IGHV1-69* in DWS+ is shown in red tones. *DWS−* dengue without warning signs; *DWS+* dengue with warning signs; *NA* not applicable

A predominant use of certain IGHV families and segments in plasmablast-derived anti-DENV antibodies has been described [16]. Relative IGHV usage frequencies are expected to be heavily influenced by IgG transcription levels according to B cell functional stage. Thus, to compare relative IGHV family and segment usage, we analyzed IGHV family and segment usage per lineage (Fig. 4a, b), and per clonotype (Additional file 7), as well as IGHV family-relative and segment-relative transcription (based on read count, regardless of clonotype or lineage composition) (Additional file 7A, B). To identify a potential bias in IGHV use during acute DENV infection, we measured the difference between the relative frequencies of each IGHV family or segment during the acute phase minus the corresponding post-convalescent phase (ΔA − Pc). Interestingly, hierarchical clustering of ΔA − Pc IGHV usage per lineage revealed two patient clusters: one that showed increased IGHV3 and decreased IGHV1 family usage, containing all patients with primary infections and two with DWS− with secondary infections; and the other showing increased IGHV1 and decreased IGHV3 family usage, containing the rest of the patients with secondary infections including all DWS+ patients (Fig. 4a). IGHV1 and IGHV3 usage was different between DWS− and DWS+ (two-way ANOVA; *p* < 0.01 and *p* < 0.001, respectively).

To identify the IGHV segments responsible for such differences, ΔA − Pc IGHV segment usage was compared. This revealed a significant increase in *IGHV1-2* (two-way ANOVA, *p* < 0.01) and *IGHV1-69* (two-way ANOVA, *p* < 0.001) usage frequency in acute DWS+ (Fig. 4b, Table 3). Further analysis of differential IGHV family or segment usage according to clonotypes consistently revealed that *IGHV1-69* was significantly increased during acute DWS+ (two-way ANOVA, *p* < 0.001). One additional segment belonging to the IGHV1 family, *IGHV1-18*, was also significantly increased during acute DWS− but not DWS+ (two-way ANOVA, *p* < 0.05) (Table 3, Additional file 7C).

**Table 3:** IGHV differential (ΔA − Pc) usage summary

Family or segment	Relative transcription		Clonotypes frequency		Lineages frequency	
	Clinical status	Immune status	Clinical status	Immune status	Clinical status	Immune status
*IGHV1*	* (NS)	* (NS)	** (NS)	NS (NS)	** (NS)	NS (NS)
*IGHV3*	* (NS)	* (NS)	*** (NS)	NS (NS)	*** (NS)	* (NS)
*IGHV1-2*	* (*)	NS (*)	NS (**)	NS (NS)	** (**)	NS (NS)
*IGHV1-18*	NS (NS)	NS (NS)	* (*)	NS (NS)	NS (NS)	NS (NS)
*IGHV1-69*	*** (NS)	NS (NS)	*** (**)	* (NS)	*** (NS)	NS (NS)
*IGHV2-5*	NS (NS)	NS (NS)	NS (***)	NS (NS)	NS (*)	NS (NS)

*p < 0.05; **p < 0.01; ***p < 0.001; *NS* not significant. Two-way ANOVA with Bonferroni correction. Values in parentheses derive from filtering out lineages with ≥ than 30 reads

The estimation of IGHV usage has limitations because it is based on a comparison between a predominantly DENV-specific plasmablast repertoire (Fig. 3b) [42], with the repertoire of a mixed IgG^+^ non-DENV-specific mB cell and plasmablast population during the post-convalescent phase (Fig. 3c). We estimated that during the post-convalescent phase, the probability of sampling an mB cell containing 30 reads or higher was very low (≤2.06447E − 06). To compare IGHV usage in plasmablasts during the acute and post-convalescent phases, we filtered out lineages below 30 reads, yielding similar results to those in the bulk analysis, but adding IGHV2-5 as significantly overrepresented (ANOVA *p* < 0.001) during the acute phase. Table 3 summarizes the analysis of differential IGHV family/segment usage in terms of the level of aggregation (lineages, clonotypes, relative expression, and filtering according to lineage size) and of differences between clinical and immune status. Because this analysis was performed using unsorted B cell populations, it is not possible to know the exact number of B cells involved. However, these results suggest a potential selection bias by DENV of B cells using segments of the IGHV1 family, particularly *IGHV1-2, IGHV1-18*, and *IGHV1-69*. Although *IGHV1-2* raw expression was significantly increased in acute DWS+ (Additional file 7B; two-way ANOVA, *p* < 0.05), no differences were found at the clonotypic frequency level (Additional file 7C). Such differences in relative IGHV transcription (raw IGHV usage) may imply different proportions of cells with high IgG versus low IgG transcription, and not differences in the number of B cells using a particular IGHV segment. Biased usage of particular IGHV segments in response to a common pathogen in different individuals suggests that recognition is highly influenced by VH regions other than CDRH3 [45]. Thus, the composition of such biased IGHV expansions should be polyclonal. Indeed, digital CDRH3 “spectra-typing” for biased IGHV segments at the lineage level confirmed this to be the case (Additional file 8).

To address if allelic variation in IGHV segment could influence the expansion of particular IGHV segments, we characterized the IGHV genotypes for *IGHV1-2*, *IGHV1-18*, and *IGHV1-69* (Additional file 9). We found no correlation between allele type and expansion in the corresponding IGHV segments.

### Lower SHM in the acute phase

**Fig. 5:**
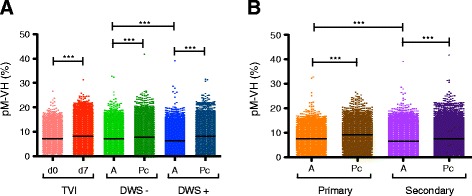
IgG B cell repertoire during acute DENV infection is associated with low somatic hypermutation (SHM) rates. SHM rates are shown according to clinical status (**a**) and according to immune status (**b**), measured as the proportion of mutations along the VH region [pM-VH (%)]. **a** The global SHM rate during the acute phase is significantly lower in acute DENV infection, but not as a result of the 2008–2009 seasonal trivalent inactivated influenza vaccination (*TVI*). **b** Global SHM rates are lower in the acute phase of DENV infection and significantly lower in acute secondary infection than in acute primary infection. Dengue without warning signs (*DWS−)* acute (*A*), *green*; DWS− post-convalescence (*Pc*), *dark green*; dengue with warning signs (*DWS+*) A, *blue*; DWS+ Pc, *dark blue*; d0 TVI, *pink*; d7 TVI, *red*; primary infection A, *orange*; primary infection *Pc*, *brown*; secondary infection A, *light purple*; secondary infection Pc, *dark purple* (Kruskal–Wallis test, Dunn’s correction for multiple testing, **p < 0.01, ***p < 0.001)

A hallmark of the adaptive humoral immune response is affinity maturation as a result of antigen re-exposure. Affinity maturation occurs by SHM and mainly affects antigen-selected GC B cells [12]. SHM is mechanistically coupled with CSR [46]. Given that the majority of the samples analyzed in this study focused on the IgG compartment from secondary infections (class switched B cells), higher levels of SHM in B cells would be expected. In order to detect whether higher levels of SHM had indeed occurred, the percentage of mutations in the IGHV, using the germ-line as reference segments, was calculated for each consensus of lineages [36]. We observed that acute DENV infection had an overall lower proportion of SHM than the corresponding level during post-convalescence, regardless of the clinical (Fig. 5a) or immune status (Fig. 5b). This effect was different from that observed in the 2008–2009 trivalent inactivated influenza vaccine (TIV), in which the proportion of SHM at 7 days post-vaccination increased (Fig. 5a) [23]. Interestingly, SHM levels were significantly lower in DWS+ than in DWS−, and in secondary than in primary DENV infection (Fig. 5b). Moreover, among acute secondary cases, lower SHM levels were found in DWS+ than in DWS− (Fig. 5a). SHM is the basis for selection of high-affinity antibodies [12]; nevertheless, calculation of non-synonymous mutations yielded the same results as overall mutation rates (Additional file 10).

In mice, marginal zone (MZ) B cell subsets are less dependent on T cell help, can be class-switched to IgG, have lower SHM rates, and have a differential IGHV usage [47], suggesting that in human DENV infection, the participation of a particular IgG^+^ B cell subset using unmutated or poorly mutated IGHV segments may take place. To determine if the reduction of SHM particularly affected certain IGHV segments during acute DENV infection, we calculated SHM rates according to IGHV segment. Significantly lower levels of SHM during acute DENV infection compared to post-convalescence were observed for *IGHV1-2*, *IGHV1-18*, and *IGHV1-69* (*p* < 0.001) (Additional file 11). As for the total repertoire, significantly lower levels of SHM of *IGHV1-2* were observed in acute DWS+ compared to acute DWS− (*p* < 0.001) and in acute secondary versus acute primary infections (Additional file 11A, B). In the case of *IGHV1-18* and *IGHV1-69*, acute secondary infection had significantly lower SHM levels than acute primary infection (Additional file 11D–F); however, no significant differences were observed between acute DWS+ and DWS− (Additional file 11).

**Fig. 6:**
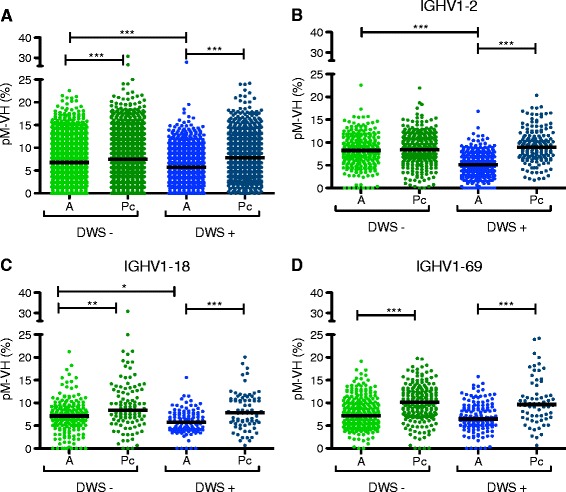
Low somatic hypermutation (SHM) rates in IgG antibody-secreting cells (ASCs) during acute DENV infection compared to steady state ASCs in post-convalescence (*Pc*). As a result of Monte Carlo simulation, we estimated that it was very unlikely for samples to have a memory B (mB) lineage larger than 30 reads during post-convalescence (*p* = 2.0645E − 06). The SHM rates were recalculated in *in silico*-sorted ASCs during post-convalescence, and classified according to clinical status. **a** Global SHM levels. **b** SHM in lineages using *IGHV1-2*. **c** SHM in lineages using *IGHV1-18*. **d** SHM in lineages using *IGHV1-69. A* acute, *DWS−* dengue without warning signs, *DWS+* dengue with warning signs

Regarding IGHV overrepresentation analysis, to avoid comparing SHM levels in a mainly DENV-specific plasmablast repertoire during the acute phase with a mixed non-DENV-specific plasmablast and non-DENV-specific mB cell repertoire during the post-convalescent phase, we filtered out all lineages with fewer than 30 reads (Additional file 1). Thus, we compared the proportion of SHM in DENV-specific plasmablasts during the acute disease with non-DENV-specific plasmablasts during the post-convalescent phase. The SHM levels were significantly lower during the acute phase, although there was no significant difference in *IGHV1-2* SHM levels in DWS− patients (Fig. 6).

To avoid potential non-independence effects imposed by sampling clonally related lineages, we also performed the same SHM estimation but instead of random lineage subsampling in each library, we randomly subsampled 250 clonotypes and performed the SHM analysis in the corresponding largest lineage. The results using this approach agreed with the lower SHM rates in the bulk analysis, indicating that our SHM estimates were not the result of sampling bias (Additional file 12).

Moreover, to discard the possibility that clonotype (CDRH3) clustering threshold identity (97 %) could artificially sub-estimate SHM levels, we performed an analysis with the reconstructed repertoire at a CDRH3 threshold identity of 92 %. Under these parameters, SHM levels were equally low during the acute phase (Additional file 13).

As shown in Figs 5 and 6a, global SHM rates were lower in acute DENV infection, suggesting that this effect is not restricted to only *IGHV1-2*, *IGHV1-18*, and *IGHV1-69*. Calculation of SHM rates for all IGHV segments during acute and post-convalescent DENV infection and for controls (subjected to hierarchical clustering according to IGHV segment) revealed significantly higher SHM rates in controls and post-convalescent individuals than in patients with acute DENV infection (Mann–Whitney U test, *p* < 0.001) (Additional file 14).

Because the number of lineages belonging to a given B cell clonotype is the result of SHM, a straightforward prediction derived from lower SHM rates is that the number of lineages per heavy chain clonotype during the acute phase of DENV infection will also be low. Consistent with our observations, the clonotype per lineage ratio (1/lineages) was significantly reduced during the acute phase of DENV infection in both DWS+ and DWS− (Kruskal–Wallis test, Dunn’s correction, *p* < 0.001).

**Fig. 7:**
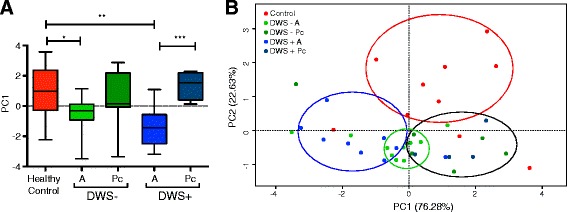
Multilevel principal component analysis of mutational parameters in acute DENV infection. **a** Single principal component plot of PC1, which represents the number of total, non-synonymous, and synonymous mutations in IGHV segments of controls (*red*), acute DWS− (*DWS− A*, *green*), post-convalescent DWS− (*DWS− Pc*, *dark green*), acute DWS+ (*DWS+ A*, *blue*), and post-convalescent DWS+ (*DWS+ Pc*, *dark blue*). Lower scores for PC1 were observed during the acute phase of DENV infection. DWS− A and DWS− Pc were significantly different (Kruskal–Wallis test, **p < 0.01). **b** Bi-plot showing clustering of patient samples according to clinical status. *DWS−* dengue without warning signs; *DWS+* dengue with warning signs

Finally, we performed a multivariate analysis based on multilevel principal component analysis to search for association patterns between SHM rates, clonal selection (lineage relative frequency), and clinical condition. We used the mean proportion (%) of all mutations, non-synonymous and synonymous mutations in the IGHV segment, as well as the mean relative frequency of 1280 randomly chosen lineages as variables for the analysis. CDRH3 length was excluded because it did not contribute significantly to variance. Two components, PC1 and PC2 explained 76.3 % and 22.6 % of the variance, respectively, with a cumulative proportion of 98.9 %. Mean PC1 score was significantly different between acute DWS+ and post-convalescence (*p* < 0.01) (Fig. 7a). Although PC1 was lower in acute DWS− than in post-convalescence, no significant differences were found. However, PC1 was significantly lower in DWS− and DWS+ than in the healthy control group (*p* < 0.05 and *p* < 0.01, respectively). Bi-plots of PC1 and PC2 showed four major clusters, one containing the majority of the healthy control samples, a second containing DWS+, a third containing most of the DWS− sample, and a fourth containing most of the post-convalescent patients, regardless of clinical status during acute disease (Fig. 7b). Taken together, mutation analysis using supervised and non-supervised approaches robustly supports that circulating IgG^+^ B cells during acute DENV infection were less hypermutated than IgG^+^ B cells in the post-convalescent phase or in healthy controls.

### Convergent CDRH3 signatures in acute dengue infection

**Fig. 8:**
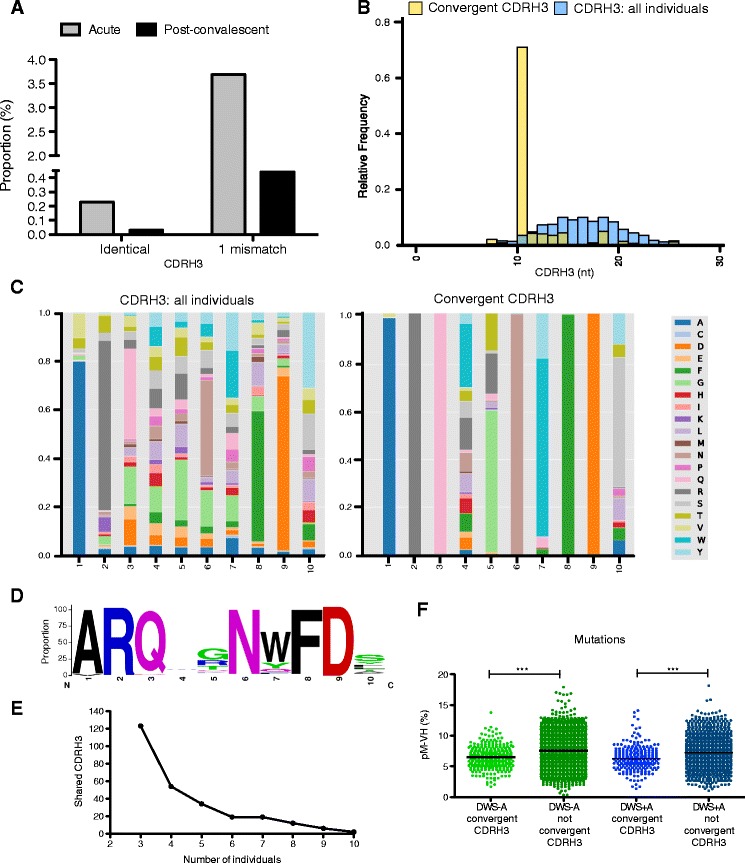
Complementarity determining region heavy 3 (*CDRH3*) signatures in acute DENV infection. A search for convergent CDRH3 signatures was performed using previously published data [41] or de novo, using the *Find_CDR3* function of *ImmunediveRsity* [30]. **a** Proportion of identical or similar (one-mismatch) CDRH3s in our acute and post-convalescent datasets compared to a set of 151 convergent CDRH3s associated with acute DENV infection previously described [41]. **b** CDRH3 length distribution of de novo identified convergent CDRH3 signatures compared to overall CDRH3 length distribution. We found 1365 clonotypes representing 269 identical CDRH3 shared in at least three individuals with acute DENV infection. A predominant 10-residue CDRH3 signature was found. **c** Amino acid residue composition in 10-residue-long CDRH3 (*left*) and in 10-residue-long de novo identified convergent CDRH3s associated with DENV infection (*right*). **d** LOGO plot of consensus 10-residue-long convergent CDRH3 signatures. **e** Absolute number of shared CDRH3s as a function of the number of individuals who share them. **f** Proportion of somatic hypermutation per lineage in convergent and non-convergent CDRH3s in acute DWS – (*green*) and DWS+ (*blue*). DWS *−* dengue without warning signs; DWS *+* dengue with warning signs

Convergent antibody signatures in different individuals responding to a variety of viral infections (reviewed in [48]), including dengue virus infection, have been described [41]. We used as a query a dataset of 151 convergent CDRH3s [41] to search for near-identical matches (one mismatch tolerance) or identical matches on our acute or post-convalescent databases. We found 1098 shared CDRH3 in at least three individuals during acute infection (19 cases) versus 53 shared CDRH3 in at least three post-convalescent individuals (10 cases). Correcting for differences in the total number of clonotypes, 3.6 % of clonotypes were shared in at least three individuals during acute infection compared to 0.44 % during post-convalescence (8.3-fold difference) (Fig. 8a). A similar approach searching for identical CDRH3s revealed that 0.23 % of clonotypes (68) were shared in at least three individuals during acute infection compared to 0.03 % (3) during post-convalescence (9.1-fold difference) (Fig. 8a). Only three convergent CDRH3s were found in no more than two individuals of a group of healthy individuals prior to vaccination with TIV (Fig. 5a).

We also searched for convergent CDRH3s regardless of their presence in the dataset described in [41] (*de novo*). We found 1365 clonotypes representing 269 identical CDRH3s shared in at least three individuals during acute infection (0.9 % of all clonotypes in acute infection). Interestingly, among shared clonotypes there was a predominant CDRH3 length of 10 residues (70 %) (Fig. 8b–e). Two CDRH3s, ARQFGNWFDS and ARQWGNWFDL, were shared in 10 individuals (10/19, 52 % of individuals) (Fig. 8e). A CDRH3, ARQ*L*GNWFD*S*, present in nine individuals was similar, although not identical to, the ARQ*I*GNWFD*P* signature described in [41] (differences in italics). Finally, we addressed whether the convergent heavy chain clonotypes were less hypermutated. We sampled the largest lineage of convergent and non-convergent VH clonotypes as in Additional file 12. As expected, lower SHM rates were found in convergent VH lineages (Fig. 8f).

## Discussion

Understanding of the immune response to DENV infection has been hampered by the lack of adequate experimental models [49], but represents an urgent goal for developing safe vaccination strategies and as the basis for understanding the role of population immunity in disease transmission dynamics [50]. Using an IgG Rep-Seq approach, we have shown that in the course of an acute DENV infection there is an increase in B cell clonal diversity in peripheral blood, presumably as a result of DENV-specific plasmablast mobilization. We also documented differences in the relative frequencies of B cells using certain IGHV segments belonging to the IGHV1 family according to clinical status. More importantly, we found a paradoxically low SHM frequency in the acute phase, as compared to the corresponding post-convalescent phase. Also paradoxical is the fact that the SHM frequency was even lower in secondary than in primary DENV infections. Interestingly, for some IGHV segments such as *IGHV1-2*, the SHM rates were lower in DWS+ compared to DWS− infection.

HTS of the B cell repertoire has been applied to explore higher order structural properties of the antibody repertoire [35, 51], track leukemia residual disease [52], uncover clonal lineages and patterns of SHM profiles in broadly neutralizing antibodies in human immunodeficiency virus [53], as well as to analyze the antibody response in influenza virus infection [54] and vaccination [23, 55, 56]. It is particularly relevant for the work presented here to explore convergent antibody signatures in DENV infection [41]. Being a relatively recently developed approach, there are substantial methodological and analytical differences by which the research groups have coped with the challenge of mining the lymphocyte repertoire complexity. An important methodological difference is whether the starting material for Ig sequencing is DNA or RNA. Sequencing DNA has the advantage of providing a single copy per B cell as template for PCR amplification, thus, assuming unbiased PCR amplification, the clonal size is a reflection of the number of clonally related B cells in a given clone. An important advantage of using RNA-derived libraries for sequencing, particularly those generated by 5′RACE-PCR, is that less PCR amplification bias is expected owing to the use of a single primer pair based on invariant amplicon flanks [20]. However, in contrast to DNA sequencing, differences in B-cell receptor (BCR) expression related to differentiation stage hamper an accurate estimation of clonal composition and size. To cope with potential distortions of Ig expression levels derived from unsorted B cells, we collapsed sequencing reads according to common clonal origin (heavy chain clonotypes) or further into lineages (consensus of sequences from a common clonal origin displaying a differential SHM pattern). Using this approach, IGHV frequency usage and SHM levels were quantified with respect to the corresponding denominator, reducing biases related to Ig expression levels.

In the context of DENV infection, we used the absolute lineage number, rarefaction analysis, and Shannon–Weaver index (entropy) as an approximation to estimate diversity in a subsample B cell repertoire (Fig. 2, Additional file 6). These analyses suggest that during acute DENV infection there is an increase in B cell diversity. An increase in B cell clonality due to clonal expansion using P[collision] was recently described [41]. We argue that our results are not contradictory to this; P[collision] is estimated by replicate sampling and measures the probability of finding clonally related B cells in the replicates. Thus, clonal expansions can be readily assessed and proved during the acute phase of DENV infection [41]. Here, because we used RNA as the starting material and sequenced a unique sample, we cannot evaluate clonal expansion directly (because clonally related sequences may be clustered during the reconstruction of clonotypes and lineages). Nevertheless, we interpret the higher species richness in rarefaction analysis and the increased Shannon–Weaver index as the result of higher numbers of circulating B cells, which could be explained, at least partially, by the massive plasmablast migration that occurs during the acute phase of the disease [15, 19, 42, 57].

Preferential IGHV usage has been described in antigen-specific B cells in models of cytopathic viral infection. In vesicular stomatitis virus (VSV) infection in mice, a rapid initial IgG-neutralizing response of defined germline unmutated IGHV segments (*VHQ52*) confer protection. Secondary challenge is associated with a shift in the use of other segments (*VH7183* and *VHJ558*) and the occurrence of SHM [58, 59].

The generation of unmutated IgG^+^-switched mB cells beyond the VSV model has been described in a model of Polyomavirus infection [60] in *Bcl6*-deficient mice, which are devoid of T_FH_ cells and thus lack a GC reaction [61, 62]. Also in mice, an extrafollicular response to *Salmonella* with sufficiently low levels of SHM to promote affinity maturation was recently described [63]. Strikingly similar results have been obtained in human Rotavirus infection, where the response to VP6 is mediated in part by class-switched mB cells exhibiting low hypermutation rates that predominantly use *IGHV1-46* [64]. These observations have been generalized as part of the concept of “natural antibodies,” which can be IgM, IgG, and IgA. These are encoded by unmutated germline antibody genes, are produced as a rapid T cell-independent response against a variety of viruses with relatively high affinity, and recognize repetitive structures such as viral capsids, but are intrinsically poly-reactive [65]. A notable example of this type are *IGHV1-69*-coded antibodies that have been recurrently implicated in recognition against influenza A, hepatitis C, and human immunodeficiency viruses [45, 48, 66–70].

**Fig. 9:**
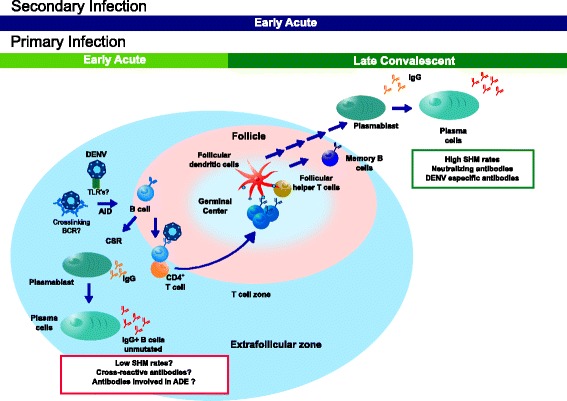
Proposed model for B cell responses in secondary lymphoid organs during DENV infection. DENV recognition by naïve B cells and during early primary infection occurs in extra-follicular regions and induces a rapid differentiation to Ig-switched antibody-secreting cells (*ASCs*) secreting unmutated poly/cross-reactive IgG. This pathway may be initiated by DENV low-affinity interactions with B cells using an “innate” IGHV segment such as *IGHV1-69* [70] that promotes receptor-mediated endocytosis and TLR7 recognition. Both signals promote T-independent activation and class switch recombination (*CSR*). Later, the classical germinal center T-dependent response develops, peaking 1–2 weeks after symptoms onset. A secondary infection with a different serotype triggers the same “natural” B cell response derived from naïve B cells that temporally overlaps with a more rapid secondary T-dependent B cell response

In accordance with a natural antibody response, DENV activates poly-reactive, natural IgG B cells after primary and secondary infection [15]. Studies with human anti-DENV monoclonal antibodies have revealed that a large proportion of DENV-reactive human antibodies are highly cross-reactive [17, 18] and recognize quaternary epitopes present only on the viral particle, but not in monomeric E protein [71]. Here we found low levels of SHM and differential usage of *IGHV1-2*, *IGHV1-18*, and *IGHV1-69* in acute DWS− and DWS+, suggesting their potential implication in natural DENV recognition, cross-reactivity, and antibody-dependent enhancement. The 5′RACE-PCR approach used for VH library generation rules out a possible bias for amplification of unmutated over hypermutated sequences. We propose that during acute primary and secondary DENV infections, at least two effector B cell differentiation pathways co-occur, one consisting of a natural poorly mutated antibody IgG response (similar to that occurring in the VSV infection model and Polyomavirus infections in mice and Rotavirus infections in humans), and the other of a classic secondary T cell- and GC-dependent B cell response pathway (Fig. 9). Although the relative contribution of both pathways to SHM levels in peripheral IgG^+^ B cells is difficult to assess, the presented evidence suggests that the GC-i response is strong enough to significantly influence the levels of SHM.

Interestingly, lower IgG SHM rates were found in acute disease, and appeared to be more accentuated in DWS+ than in DWS−, and in secondary more than in primary acute infections. This effect was particularly clear in *IGHV1-2*-expressing IgG B cells. Consistent with its role in natural antibody recognition, *IGHV1-2* is frequently used by neonatal cord blood IgM^+^ lymphocytes [72] and in MZ B cell lymphomas [73, 74], which are thought to derive from chronic pathogen-mediated selection [75]. MZ B cells are capable of mounting class-switched and class-unswitched T-independent as well as T-dependent B cell responses [47]. Normal MZ B cells produce rapid responses to blood-borne pathogens and could be the source of natural IgM and IgG responses against DENV. However, no modification in MZ B cells was observed in children with acute DENV infection [19]. Moreover, human MZ B cells are usually hypermutated [47], thus suggesting that during acute DENV infection, MZ B cells are not the source of circulating poorly mutated IgG^+^ B cells.

Among other limitations of the HTS approach is that our VH libraries derived from total peripheral blood IgG-expressing B cells; as such, we have no information regarding the relative contribution of different B cell subsets or their antigen specificity. However, in support of our results, a preferential use of the IGHV1 family in plasmablasts, but not mB cells, has been described in acute DENV infection [16]. Moreover, the high proportion of DENV-specific plasmablasts occurring during acute infection [42] provides further support to the claim that the observed repertoire differences may be DENV-specific as well.

Another limitation of working with unsorted IgG^+^ B cells, namely mB cells and ASCs (plasmablasts and plasma cells) is that their relative proportions vary dramatically during acute dengue infection, making it difficult to compare their respective repertoires. We used a Monte Carlo simulation method to estimate the probability of sampling either subpopulation depending on the relative proportions of mB cells and ASCs. A limitation of this simulation is that it cannot discriminate ASC from mB cells that have proliferated extensively. However, the lack of variation in the proportion of mB cells in acute dengue argues against this possibility [19, 57]. Interestingly, we estimated that the probability of sampling mB cells during acute dengue is negligible owing to the large number of plasmablasts and their corresponding high Ig expression levels. Likewise, during the post-convalescence phase, the probability of sampling an mB cell larger than 30 reads is negligible as well. This approach allowed us to partition the repertoire according to Ig expression level and to confirm our observations regarding IGHV usage and SHM level differences during acute disease. These experiments also highlight an additional advantage of using 5′RACE-PCR for Rep-Seq.

We are beginning to elucidate the molecular basis for T-independent CSR to IgG. In mice, TLR7 and TLR9 synergize with BCR signaling to promote activation-induced cytidine deaminase expression, which is required for CSR and SHM [76]. Although both processes are functionally coupled, it is not clear how SHM is prevented in B cells undergoing T-independent CSR. TLR-mediated signaling pathways are implicated in promoting T-independent CSR [76] and T-independent IgG responses against Polyomavirus require MyD88 [77]. Endosomal DENV recognition by TLR7 could provide a synergic signal with the BCR for T-independent CSR anti-DENV cross-reactive B cells (Fig. 9).

The proposed model of extra-follicular B cell responses offers a potential explanation for the production of low-affinity sub-neutralizing and cross-reactive IgG antibodies that may promote disease enhancement. Thus, in the case of DENV vaccination and analogously to observations in Polyomavirus-immunized mice [60], strategies that promote T-independent B cell responses (i.e., TLR7 agonist) could be associated with a higher risk of severe disease upon reinfection than strategies that do not [78, 79].

## Conclusions

Our results in B cell repertoire mining suggest that during acute dengue infection, in parallel with a GC-dependent pathway, a GC-independent effector differentiation pathway may occur, which manifests as preferential IGHV gene use and low SHM in IgG class-switched B cells. This non-GC pathway may not be restricted to primary infections, given that SHM levels were even lower in secondary acute infections and was more striking in acute DWS+. Further research is required to obtain definitive proof of a GC-independent B cell differentiation in response to dengue infection, as well as to define the role of poorly mutated IgG in terms of poly-reactivity and ADE. Nevertheless, our findings are relevant for understanding of the immune response to DENV and future vaccine development.

## Additional files

Additional file 1:
**Monte Carlo simulation to calculate the probability to sample mB or ASCs.** A detailed description of how the simulation model was built, including the sources of information regarding relative amounts of the relevant subpopulations (IgG^+^ mB cells and ASCs), as well as the relative Ig transcription levels in either subpopulation. (PDF 701 kb) Additional file 2:
**Clonotype and lineage clustering by** ***ImmunediveRsity*** **.** Raw IgG HTS data was subjected to repertoire reconstruction and analysis using *ImmunediveRsity*. Briefly, *ImmunediveRsity* assigns IGHV gene to each sequence and clusters them according to IGH clonotypes, which are composed of reads with the same IGHV-IGHJ rearrangement and quasi-identical CDRH3 (≥97 % identity at the nucleotide level). Within a clonotype, one or more lineages can be defined based on SHM pattern (99.5 % identity). IGHV usage frequency can thus be expressed as the proportion of reads using a particular IGHV segment (relative transcription), regardless of clonotype origin, or can be expressed as the proportion of IGH clonotypes or lineages using a particular IGHV segment regarding the total amount of clonotypes or lineages, respectively. (PDF 35 kb) Additional file 3:
**Days after onset of clinical illness for blood sampling according to clinical status.** DWS- A, *green*; DWS− Pc, *dark green*; DWS+ A, *blue*; DWS+ Pc, *dark blue*. (One-way ANOVA, *p* > 0.05, not significant). (PDF 16 kb) Additional file 4:
**Neutralization summary of DENV RVPs by human sera.** The 50 % neutralization titers (NT_50_) are shown as reciprocal serum dilutions. (XLS 29 kb) Additional file 5:
**Reads, clonal groups, and lineages numbers per individual and condition.** The table shows the number of raw and used sequences as well as the number of clonotypes and lineages per individual. (XLS 30 kb) Additional file 6:
**Higher clonal diversity during acute phase than in the post-convalescent phase, regardless of clinical classification.** Rarefaction analysis of acute vs. post-convalescent samples according to clinical status, (**A**) DWS− (A, *green* and Pc, *dark green*) and (**B**) DWS+ (A, *blue* and Pc, *dark blue*); and immune status, (**C**) primary (A, *orange* and Pc, *brown*) and (**D**) secondary infection (A, *light purple* and Pc, *dark purple*) (two-way ANOVA with Bonferroni correction, **p* < 0.05; ***p* < 0.01). Shannon–Weaver entropy values for heavy chain clonotypes according to (**E**) clinical status and (**F**) immune status and for heavy chains lineages according to (**G**) clinical status and (**H**) immune status. (DWS− A, *green*; DWS− Pc, *dark green*; DWS+ A, *blue*; DWS+ Pc, *dark blue*; primary infection acute, *orange*; primary infection Pc, *brown*; secondary infection acute, *light purple*; secondary infection Pc, *dark purple*) (For E–H, one-way ANOVA with Bonferroni correction, **p* < 0.05; ***p* < 0.01). (PDF 78 kb) Additional file 7:
**Preferential IGHV transcription and usage during acute DENV infection.** (**A**). Heat map of hierarchical clustering of the difference between acute minus post-convalescent read relative frequency (ΔA − Pc) per IGHV family. Over-transcription of IGHV family during acute phase is indicated in *yellow* tones, whereas *blue* tones indicate under-transcription. Upper rows classify patients according to clinical status (DWS−, *green* and DWS+, *blue*) and immune status (primary, *orange* and secondary, *purple*) (two-way ANOVA, Bonferroni correction for multiple testing, **p* < 0.05). (**B**) Relative transcription difference between acute minus post-convalescent (ΔA − Pc) according to IGHV segment and clinical status. *IGHV1-2* and *IGHV1-69* are overexpressed in acute DWS+ (two-way ANOVA, Bonferroni correction for multiple testing, **p* < 0.05). (**C**) Difference between the relative IGHV segment use per clonotypes (*rows*) in acute minus post-convalescent (ΔA − Pc) according to clinical status (*columns*: DWS−, *green* and DWS+, *blue*) and immune status (primary, *orange* and secondary, *purple*). Higher frequency of clonotypes using *IGHV1-18* and *IGHV1-69* in DWS+ is shown in red tones (two-way ANOVA, Bonferroni correction for multiple testing, **p* < 0.05). (PDF 131 kb) Additional file 8:
**Digital CDRH3 “spectratyping” of acute phase DENV infection.** CDRH3 “spectratyping” of all segments and according to *IGHV1-2*, *IGHV1-18*, and *IGHV1-69* segments based on the CDRH3 size of 1280 lineage subsample. Clinical and immune status is shown in upper bars. CDRH3 length (bp) is shown in *x* axis and lineage relative frequency is shown in *y* axis (*color bars*: DWS−, *green* and DWS+, *blue.* Upper lines: DWS−, *green*; DWS+, *blue;* primary, *orange*; secondary, *purple*). (PDF 619 kb) Additional file 9:
***IGHV*** **allelic variation in patients with DENV infection.** *ImmunediveRsity* output data in raw reads were used for allele calling. The matrix depicts each individual and the presence (*dark blue*) of absence (*gray*) of *IGHV1-2*, *IGHV1-18*, *IGHV1-69*, and *IGHV2-5* alleles. (PDF 22 kb) Additional file 10:
**Absolute number of non-synonymous mutations.** Quantitative analysis of non-synonymous somatic hypermutation (DWS− A, *green*; DWS− Pc, *dark green*; DWS+ A, *blue*; DWS+ Pc, *dark blue*) (Kruskal–Wallis test for non-parametric data with Dunn’s multiple comparison test, *** *p* < 0.001). (PDF 1967 kb) Additional file 11:
**Somatic hypermutation rates in selected IGHV segments according to clinical and immune status.** SHM rates are shown according to clinical status (A, C, and E) and according to immune status (B, D, and F) measured as the proportion of mutations along the VH region [pM-VH (%)]. (**A**) SHM rates of *IGHV1-2* regarding clinical status and (**B**) immune status. (**C**) SHM rates of *IGHV1-18* regarding clinical status and (**D**) immune status. (**E**) SHM rates of *IGHV1-69* regarding clinical status and (**F**) immune status. (DWS− A, *green*; DWS− Pc, *dark green*; DWS+ A, *blue*; DWS+ Pc, *dark blue*; primary infection acute, *orange*; primary infection Pc, *brown*; secondary infection acute, *light purple*; secondary infection Pc, *dark purple*) (Kruskal–Wallis test, Dunn’s correction for multiple testing, ***p* < 0.01, ****p* < 0.001). (PDF 886 kb) Additional file 12:
**SHM rates in global and selected IGHV segments of randomly subsampled clonotypes.** Each dot represents the percentage of mutations in the largest lineage of 250 randomly sampled clonotypes per individual, classified according to clinical status. (**A**) Global mutation rates; (**B**) *IGHV1-2*; (**C**) *IGHV1-18*; and (**D**) *IGHV1-69* (DWS− A, *green*; DWS− Pc, *dark green*; DWS+ A, *blue*; DWS+ Pc, *dark blue*) (Kruskal–Wallis test, Dunn’s correction for multiple testing, ***p* < 0.01, ****p* < 0.001). (PDF 483 kb) Additional file 13:
**SHM rates in global and selected IGHV segments in the** ***ImmunediveRsity*** **-reconstructed repertoire using a CDRH3 identity clustering threshold of 92 %.** Each dot represents the percentage of mutations in randomly sampled lineages per individual, classified according to clinical status. (**A**) Global mutation rates; (**B**) *IGHV1-2*; (**C**) *IGHV1-18*; and (**D**) *IGHV1-69* (DWS− A, *green*; DWS− Pc, *dark green*; DWS+ A, *blue*; DWS+ Pc, *dark blue*) (Kruskal–Wallis test, Dunn’s correction for multiple testing, ***p* < 0.01, ****p* < 0.001). (PDF 2177 kb) Additional file 14:
**Heat map representation of SHM according to IGHV segment (** ***rows*** **) and clinical status (** ***columns*** **).** Acute DENV infection had significantly lower levels of SHM than post-convalescent and control samples (Mann–Whitney U test for non-parametric data, *** *p* < 0.001). (PDF 43 kb)

## Abbreviations

ADE: antibody-dependent enhancement
ANOVA: analysis of variance
ASC: antibody-secreting cell
BCR: B cell receptor
CDRH3: complementarity determining region heavy 3
CSR: class switch recombination
DENV: Dengue virus
DWS−: dengue without warning sings
DWS+: dengue with warning sings
ELISA: enzyme-linked immunosorbent assay
GC: germinal center
HTS: high-throughput sequencing
mB: memory B cell
PC: principal component
pM-VH: proportion of mutations in VH region
RACE: rapid amplification of cDNA ends
Rep-Seq: Repertoire Sequencing
RT-PCR: reverse transcriptase polymerase chain reaction
RVPs: reporter virus particles
SHM: somatic hypermutation
TIV: trivalent inactivated influenza vaccine
VSV: vesicular stomatitis virus

## References

[1] Halstead SB (2007). Dengue. The Lancet.

[2] WHO (2009). Dengue: guidelines for diagnosis, treatment, prevention and control.

[3] Bhatt S, Gething PW, Brady OJ, Messina JP, Farlow AW, Moyes CL (2013). The global distribution and burden of dengue. Nature.

[4] Wu SJ, Grouard-Vogel G, Sun W, Mascola JR, Brachtel E, Putvatana R (2000). Human skin Langerhans cells are targets of dengue virus infection. Nat Med.

[5] Durbin AP, Vargas MJ, Wanionek K, Hammond SN, Gordon A, Rocha C (2008). Phenotyping of peripheral blood mononuclear cells during acute dengue illness demonstrates infection and increased activation of monocytes in severe cases compared to classic dengue fever. Virology.

[6] Ho LJ, Wang JJ, Shaio MF, Kao CL, Chang DM, Han SW (2001). Infection of human dendritic cells by dengue virus causes cell maturation and cytokine production. J Immunol.

[7] Wahala WM, Silva AM (2011). The human antibody response to dengue virus infection. Viruses.

[8] Guzman MG, Alvarez M, Halstead SB (2013). Secondary infection as a risk factor for dengue hemorrhagic fever/dengue shock syndrome: an historical perspective and role of antibody-dependent enhancement of infection. Arch Virol.

[9] Halstead SB (2009). Antibodies determine virulence in dengue. Ann N Y Acad Sci..

[10] Maul RW, Gearhart PJ (2010). Controlling somatic hypermutation in immunoglobulin variable and switch regions. Immunol Res.

[11] Xu Z, Zan H, Pone EJ, Mai T, Casali P (2012). Immunoglobulin class-switch DNA recombination: induction, targeting and beyond. Nat Rev Immunol.

[12] Victora GD, Nussenzweig MC (2012). Germinal centers. Annu Rev Immunol..

[13] Tarlinton D, Good-Jacobson K (2013). Diversity among memory B cells: origin, consequences, and utility. Science.

[14] Midgley CM, Bajwa-Joseph M, Vasanawathana S, Limpitikul W, Wills B, Flanagan A (2011). An in-depth analysis of original antigenic sin in dengue virus infection. J Virol.

[15] Balakrishnan T, Bela-Ong DB, Toh YX, Flamand M, Devi S, Koh MB (2011). Dengue virus activates polyreactive, natural IgG B cells after primary and secondary infection. PLoS One.

[16] Xu M, Hadinoto V, Appanna R, Joensson K, Toh YX, Balakrishnan T (2012). Plasmablasts generated during repeated dengue infection are virus glycoprotein-specific and bind to multiple virus serotypes. J Immunol.

[17] Beltramello M, Williams KL, Simmons CP, Macagno A, Simonelli L, Quyen NT (2010). The human immune response to Dengue virus is dominated by highly cross-reactive antibodies endowed with neutralizing and enhancing activity. Cell Host Microbe.

[18] Smith SA, Zhou Y, Olivarez NP, Broadwater AH, de Silva AM, Crowe JE (2012). Persistence of circulating memory B cell clones with potential for dengue virus disease enhancement for decades following infection. J Virol.

[19] Zompi S, Montoya M, Pohl MO, Balmaseda A, Harris E (2012). Dominant cross-reactive B cell response during secondary acute dengue virus infection in humans. PLoS Negl Trop Dis.

[20] Benichou J, Ben-Hamo R, Louzoun Y, Efroni S (2012). Rep-Seq: uncovering the immunological repertoire through next-generation sequencing. Immunology.

[21] Georgiou G, Ippolito GC, Beausang J, Busse CE, Wardemann H, Quake SR (2014). The promise and challenge of high-throughput sequencing of the antibody repertoire. Nat Biotechnol.

[22] Hurtado-Diaz M, Riojas-Rodriguez H, Rothenberg SJ, Gomez-Dantes H, Cifuentes E (2007). Short communication: impact of climate variability on the incidence of dengue in Mexico. Trop Med Int Health.

[23] Cortina-Ceballos B, Godoy-Lozano EE, Tellez-Sosa J, Ovilla-Munoz M, Samano-Sanchez H, Aguilar-Salgado A (2015). Longitudinal analysis of the peripheral B cell repertoire reveals unique effects of immunization with a new influenza virus strain. Genome medicine.

[24] InDRE. Lineamientos para la vigilancia por laboratorio de Dengue. 2012. http://www.epidemiologia.salud.gob.mx/doctos/sinave/ve_lab/LINEAMIENTOS_DENGUE_2012.pdf. Accessed 18 Feb 2016 .

[25] Sd S. NORMA Oficial Mexicana NOM-032-SSA2-2002, Para la vigilancia epidemiológica, prevención y control de enfermedades transmitidas por vector. 2003. http://www.salud.gob.mx/unidades/cdi/nom/032ssa202.html. Accessed 18 Feb 2016 .

[26] Mattia K, Puffer BA, Williams KL, Gonzalez R, Murray M, Sluzas E (2011). Dengue reporter virus particles for measuring neutralizing antibodies against each of the four dengue serotypes. PLoS One.

[27] Margulies M, Egholm M, Altman WE, Attiya S, Bader JS, Bemben LA (2005). Genome sequencing in microfabricated high-density picolitre reactors. Nature.

[28] Halpern EF, Weinstein MC, Hunink MG, Gazelle GS (2000). Representing both first- and second-order uncertainties by Monte Carlo simulation for groups of patients. Med Decis Making.

[29] Kelley DE, Perry RP (1986). Transcriptional and posttranscriptional control of immunoglobulin mRNA production during B lymphocyte development. Nucleic Acids Res.

[30] Cortina-Ceballos B, Godoy-Lozano EE, Samano-Sanchez H, Aguilar-Salgado A, Velasco-Herrera MD, Vargas-Chavez C (2015). Reconstructing and mining the B cell repertoire with ImmunediveRsity. MAbs.

[31] R Development Core Team. R: A language and environment for statistical computing. Version 3.0. 2. Vienna, Austria: R Foundation for Statistical Computing; 2013.

[32] Ye J, Ma N, Madden TL, Ostell JM (2013). IgBLAST: an immunoglobulin variable domain sequence analysis tool. Nucleic Acids Res..

[33] Jost L (2006). Entropy and diversity. Oikos.

[34] Robert L, Tsoi J, Wang X, Emerson R, Homet B, Chodon T (2014). CTLA4 blockade broadens the peripheral T-cell receptor repertoire. Clin Cancer Res.

[35] Weinstein JA, Jiang N, White RA, Fisher DS, Quake SR (2009). High-throughput sequencing of the zebrafish antibody repertoire. Science.

[36] Li S, Lefranc MP, Miles JJ, Alamyar E, Giudicelli V, Duroux P (2013). IMGT/HighV QUEST paradigm for T cell receptor IMGT clonotype diversity and next generation repertoire immunoprofiling. Nat Commun..

[37] Eisen MB, Spellman PT, Brown PO, Botstein D (1998). Cluster analysis and display of genome-wide expression patterns. Proc Natl Acad Sci U S A.

[38] Zipunnikov V, Caffo B, Yousem DM, Davatzikos C, Schwartz BS, Crainiceanu C (2011). Functional principal component model for high-dimensional brain imaging. Neuroimage.

[39] Husson F, Josse J, Le S, Mazet J. FactoMineR: Multivariate Exploratory Data Analysis and Data Mining with R. R package version. 1.27 2014.https://cran.r-project.org/web/packages/FactoMineR/index.html. Accessed 18 Feb 2016.

[40] Dinno A. dunn.test: Dunn’s test of multiple comparisons using rank sums. R package version 1.2.2 2014. https://cran.r-project.org/web/packages/dunn.test/index.html. Accessed 18 Feb 2016.

[41] Parameswaran P, Liu Y, Roskin KM, Jackson KK, Dixit VP, Lee JY (2013). Convergent antibody signatures in human dengue. Cell Host Microbe.

[42] Wrammert J, Onlamoon N, Akondy RS, Perng GC, Polsrila K, Chandele A (2012). Rapid and massive virus-specific plasmablast responses during acute dengue virus infection in humans. J Virol.

[43] Morbach H, Eichhorn EM, Liese JG, Girschick HJ (2010). Reference values for B cell subpopulations from infancy to adulthood. Clin Exp Immunol.

[44] Mei HE, Yoshida T, Sime W, Hiepe F, Thiele K, Manz RA (2009). Blood-borne human plasma cells in steady state are derived from mucosal immune responses. Blood.

[45] Ekiert DC, Bhabha G, Elsliger MA, Friesen RH, Jongeneelen M, Throsby M (2009). Antibody recognition of a highly conserved influenza virus epitope. Science.

[46] Hwang JK, Alt FW, Yeap LS. Related mechanisms of antibody somatic hypermutation and class switch recombination. Microbiol Spectr. 2015;3(1). doi:10.1128/microbiolspec.MDNA3-0037-2014. 10.1128/microbiolspec.MDNA3-0037-2014 PMC4481323 26104555

[47] Cerutti A, Cols M, Puga I (2013). Marginal zone B cells: virtues of innate-like antibody-producing lymphocytes. Nat Rev Immunol.

[48] Henry Dunand CJ, Wilson PC. Restricted, canonical, stereotyped and convergent immunoglobulin responses. Philos Trans R Soc Lond B Biol Sci. 2015;370(1676). doi:10.1098/rstb.2014.0238. 10.1098/rstb.2014.0238 PMC4528415 26194752

[49] Zompi S, Harris E (2012). Animal models of dengue virus infection. Viruses.

[50] Andraud M, Hens N, Marais C, Beutels P (2012). Dynamic epidemiological models for dengue transmission: a systematic review of structural approaches. PLoS One.

[51] Mora T, Walczak AM, Bialek W, Callan CG (2010). Maximum entropy models for antibody diversity. Proc Natl Acad Sci U S A.

[52] Boyd SD, Marshall EL, Merker JD, Maniar JM, Zhang LN, Sahaf B (2009). Measurement and clinical monitoring of human lymphocyte clonality by massively parallel V-D-J pyrosequencing. Sci Transl Med.

[53] Wu X, Zhou T, Zhu J, Zhang B, Georgiev I, Wang C (2011). Focused evolution of HIV-1 neutralizing antibodies revealed by structures and deep sequencing. Science.

[54] Krause JC, Tsibane T, Tumpey TM, Huffman CJ, Briney BS, Smith SA (2011). Epitope-specific human influenza antibody repertoires diversify by B cell intraclonal sequence divergence and interclonal convergence. J Immunol.

[55] Jiang N, He J, Weinstein JA, Penland L, Sasaki S, He XS (2013). Lineage structure of the human antibody repertoire in response to influenza vaccination. Sci Transl Med.

[56] Jackson KJ, Liu Y, Roskin KM, Glanville J, Hoh RA, Seo K (2014). Human responses to influenza vaccination show seroconversion signatures and convergent antibody rearrangements. Cell Host Microbe.

[57] Garcia-Bates TM, Cordeiro MT, Nascimento EJ, Smith AP, de Melo KMS, McBurney SP (2013). Association between magnitude of the virus-specific plasmablast response and disease severity in dengue patients. J Immunol.

[58] Kalinke U, Bucher EM, Ernst B, Oxenius A, Roost HP, Geley S (1996). The role of somatic mutation in the generation of the protective humoral immune response against vesicular stomatitis virus. Immunity.

[59] Kalinke U, Oxenius A, Lopez-Macias C, Zinkernagel RM, Hengartner H (2000). Virus neutralization by germ-line vs. hypermutated antibodies. Proc Natl Acad Sci U S A.

[60] Szomolanyi-Tsuda E, Le QP, Garcea RL, Welsh RM (1998). T-cell-independent immunoglobulin G responses in vivo are elicited by live-virus infection but not by immunization with viral proteins or virus-like particles. J Virol.

[61] Toyama H, Okada S, Hatano M, Takahashi Y, Takeda N, Ichii H (2002). Memory B cells without somatic hypermutation are generated from Bcl6-deficient B cells. Immunity.

[62] Kaji T, Ishige A, Hikida M, Taka J, Hijikata A, Kubo M (2012). Distinct cellular pathways select germline-encoded and somatically mutated antibodies into immunological memory. J Exp Med.

[63] Di Niro R, Lee SJ, Vander Heiden JA, Elsner RA, Trivedi N, Bannock JM (2015). Salmonella infection drives promiscuous B cell activation followed by extrafollicular affinity maturation. Immunity.

[64] Tian C, Luskin GK, Dischert KM, Higginbotham JN, Shepherd BE, Crowe JE (2008). Immunodominance of the VH1-46 antibody gene segment in the primary repertoire of human rotavirus-specific B cells is reduced in the memory compartment through somatic mutation of nondominant clones. J Immunol.

[65] Hangartner L, Zinkernagel RM, Hengartner H (2006). Antiviral antibody responses: the two extremes of a wide spectrum. Nat Rev Immunol.

[66] Lingwood D, McTamney PM, Yassine HM, Whittle JR, Guo X, Boyington JC (2012). Structural and genetic basis for development of broadly neutralizing influenza antibodies. Nature.

[67] Sasso EH, Ghillani P, Musset L, Piette JC, Cacoub P (2001). Effect of 51p1-related gene copy number (V1-69 locus) on production of hepatitis C-associated cryoglobulins. Clin Exp Immunol.

[68] Breden F, Lepik C, Longo NS, Montero M, Lipsky PE, Scott JK (2011). Comparison of antibody repertoires produced by HIV-1 infection, other chronic and acute infections, and systemic autoimmune disease. PLoS One.

[69] Warter L, Appanna R, Fink K (2012). Human poly- and cross-reactive anti-viral antibodies and their impact on protection and pathology. Immunol Res.

[70] Lerner RA (2011). Rare antibodies from combinatorial libraries suggests an S.O.S. component of the human immunological repertoire. Mol Biosyst.

[71] de Alwis R, Smith SA, Olivarez NP, Messer WB, Huynh JP, Wahala WM (2012). Identification of human neutralizing antibodies that bind to complex epitopes on dengue virions. Proc Natl Acad Sci U S A.

[72] Prabakaran P, Chen W, Singarayan MG, Stewart CC, Streaker E, Feng Y (2011). Expressed antibody repertoires in human cord blood cells: 454 sequencing and IMGT/HighV-QUEST analysis of germline gene usage, junctional diversity, and somatic mutations. Immunogenetics.

[73] Bikos V, Darzentas N, Hadzidimitriou A, Davis Z, Hockley S, Traverse-Glehen A (2012). Over 30 % of patients with splenic marginal zone lymphoma express the same immunoglobulin heavy variable gene: ontogenetic implications. Leukemia.

[74] Warsame AA, Aasheim HC, Nustad K, Troen G, Tierens A, Wang V (2011). Splenic marginal zone lymphoma with VH1-02 gene rearrangement expresses poly- and self-reactive antibodies with similar reactivity. Blood.

[75] Bikos V, Stalika E, Baliakas P, Darzentas N, Davis Z, Traverse-Glehen A (2012). Selection of antigen receptors in splenic marginal-zone lymphoma: further support from the analysis of the immunoglobulin light-chain gene repertoire. Leukemia.

[76] Pone EJ, Zhang J, Mai T, White CA, Li G, Sakakura JK (2012). BCR-signalling synergizes with TLR-signalling for induction of AID and immunoglobulin class-switching through the non-canonical NF-kappaB pathway. Nat Commun..

[77] Raval FM, Mishra R, Garcea RL, Welsh RM, Szomolanyi-Tsuda E (2013). Long-lasting T cell-independent IgG responses require MyD88-mediated pathways and are maintained by high levels of virus persistence. mBio.

[78] Coller BA, Clements DE, Bett AJ, Sagar SL, Ter Meulen JH (2011). The development of recombinant subunit envelope-based vaccines to protect against dengue virus induced disease. Vaccine.

[79] Zhang S, Liang M, Gu W, Li C, Miao F, Wang X (2011). Vaccination with dengue virus-like particles induces humoral and cellular immune responses in mice. Virol J..

