# Supplementary material for: Lower IgG somatic hypermutation rates during acute dengue virus infection is compatible with a germinal center-independent B cell response
Source: Genome Med. 2016 Feb 25;8:23. doi: 10.1186/s13073-016-0276-1 (PMC4766701; doi:10.1186/s13073-016-0276-1)
Supplement: Additional file 1: — Monte Carlo simulation to calculate the probability to sample mB or ASCs. A detailed description of how the simulation model was built, including the sources of information regarding relative amounts of the relevant subpopulations (IgG+ mB cells and ASCs), as well as the relative Ig transcription levels in either subpopulation. (PDF 701 kb) [file 13073_2016_276_MOESM1_ESM.pdf]

Probability calculation method using second-order Monte Carlo simulation:

The model implemented in Python 2.7 assumes that there are only two IgG expressing cell types: antibody secreting cells (ASC's) and memory B cells (mB). To estimate the relative amount of Ig transcripts (or reads) from mB cells present in a peripheral blood sample, we designed a computational protocol consisting in a second-order Monte Carlo simulation [1], which integrates the following stages: In the first step, the protocol samples a population of 500 individuals to estimate the probability of picking a mB cell accounting for individual variation (normal distribution, 5% variance). The probabilities were evaluated in different scenarios with variable proportions of mB cells, so that these distributions were calculated starting from 99% of mB cells (complement of ASC, 1%) and decreased down to 1% of mB cells (complement of ASC, 99%). In the second step, the Ig transcript distribution of mB cells or ASC is sampled to estimate the expected amount of Ig transcription in each cell. Ig transcript distributions were kept fixed in all mB to ASC ratios.

In the third step, a loop of length equivalent to the amount of cells in the sample (i.e. cell by cell calculation) is introduced. As the probability of finding a mB cell or a ASC in the sample is already know from the previous step, depending on the sampled cell in each loop, either one of two distributions of Ig transcripts (reads) corresponding to mB cells or ASC is sampled. At the end of the loop, the relative amount of transcript present in the sample according to its cell type origin is known, such that the probability of observing the transcripts from either cell type can be calculated. The probability of observing a transcript belonging to a mB cell or a ASC can be deduced when the loop finishes as:

$$Probability\ of\ observing\ a\ transcript\ of\ the\ n-th\ mB\ cell\ = \frac{number\ of\ transcripts\ expresed\ by\ the\ n-th\ mB\ cell}{number\ of\ transcripts\ in\ all\ the\ sample}$$

$$Probability\ of\ observing\ a\ transcript\ of\ the\ m-th\ ASC\ = \frac{number\ of\ transcripts\ expresed\ by\ the\ m-th\ ASC}{number\ of\ transcripts\ in\ all\ the\ sample}$$

A critical parameter is the relative Ig expression level in ASC and mB cells. Plasma cells express 100-fold more Ig transcripts that resting B cells [2]. However, plasmablasts are in a transitional stage towards a plasma cell, and we do not expect they express Ig in the same amount as a plasma cells. We searched the Human Body Map 2.0 project blood RNA-seq alignments (file: GRCh38.illumina.blood.1.bam) (http://www.ensembl.org/Homo_sapiens/Info/Index), to calculate relative transcription of surface IgG (mB cells) and

soluble IgG (ASC). Estimation of total Ig transcription was based on the average expression levels (peak height score) of the CH1, CH2 and CH3 exons of IGHG1, IGHG2, IGHG3 and IGHG4 (Figure 1). Similarly, relative transcription of surface IgG was estimated by averaging peak height score for the transmembrane and intracellular domain coding exons.

Figure 1

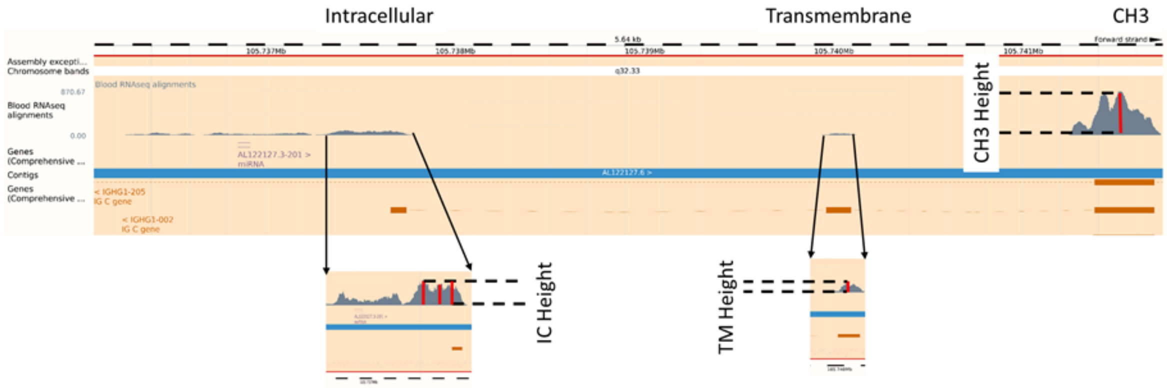

Finally, secreted IgG transcription was calculated by subtracting the average relative transcription levels of the transmembrane and intracellular domains coding exons. The anchored Ig/secreted Ig ratio was estimated 1:12. Relative transcription levels for the intracellular, transmembrane and CH exons are shown in the following table:

IgG transcription in Peripheral blood. RNA-seq data*

IgG	CH1	CH2	CH3	Total Ig mean	Secreted IgG	Transmembrane exon	intracellular exon			Anchored Ig mean	Secreted/anchored ratio
**Subtypes**						A	B	C	D		
IgG1	493	607	860	653	587	31	85	67	81	66	8.9
IgG2	475	631	1,157	754	701	36	60	63		53	13.2
IgG3	197	159	402	253	225	43	26	16	26	28	8.1
IgG4	195	220	425	280	267	31	14	3	5	13	20.1
**Total**	**1,360**	**1,617**	**2,844**	**1,940**	**1,794**	**141**	**185**	**149**	**112**	**147**	**12.6**

* Human Body Map 2.0 project.

Thus, mB cells express 100 arbitrary units (a.u) (under a normal distribution, 5% variance), whereas it is clear that the probability of observing *n* reads of the same cell goes asymptotically to zero. To establish a cutoff, we proceed calculating the probability of

observing *n* reads of the same cell directly from the probabilistic simulation of the events. To calculate the probability of observing the same cell multiple times, the algorithm proceeds as follows: If the transcript is observed, we achieve one read for this lineage and the procedure is repeated with the same conditions to determine if a second transcript can be observed, if it does, a second read is achieved and the procedure is repeated again. This internal loop ends when the event of observing the read in the *n*-th is zero.

Finally, the probability of observing a transcript belonging to a mB or ASC cell in a sample is calculated averaging over both cells types and per read. The process was repeated 500 times and 500 average values were obtained simulating 500 samples of 500 individuals. The probability distribution in acute dengue and post-convalescence is shown:

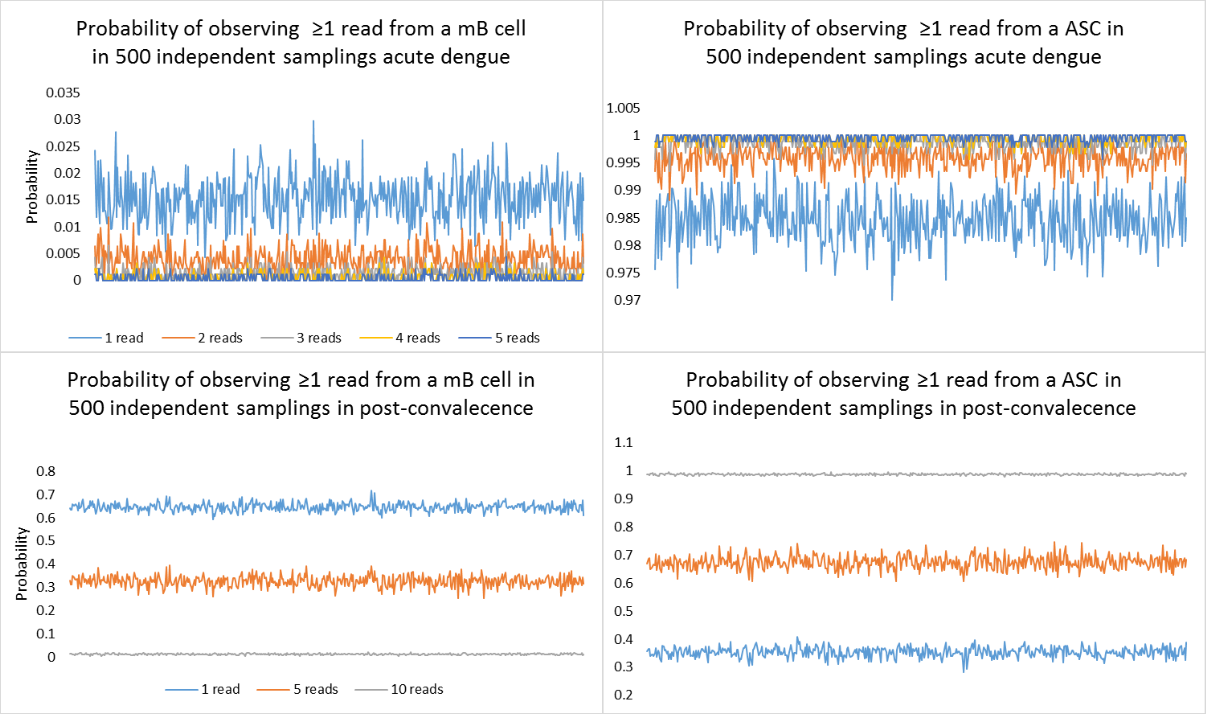

**Proportion of memory cells in the sample**	*0.99*	*0.97*	*0.95*	*0.9*	*0.8*	*0.6*	*0.4*
**Probability of observing transcripts from mB cells**							
1 read	0.942	0.725	0.582	0.310	0.122	0.035	0.007
2 reads	0.927	0.669	0.478	0.188	0.051	0.010	0.000
3 reads	0.911	0.617	0.384	0.113	0.023	0.004	0.000
4 reads	0.892	0.656	0.305	0.069	0.011	0.002	0.000
5 reads	0.871	0.444	0.240	0.042	0.006	0.001	0.000
10 reads	0.776	0.238	0.053	0.000	0.000	0.000	0.000
20 reads	0.603	0.061	0.003	0.000	0.000	0.000	0.000
30 reads	0.468	0.015	0.000	0.000	0.000	0.000	0.000
**Probability of observing transcripts from ASC's**							
1 read	0.058	0.275	0.418	0.690	0.878	0.965	0.993
2 reads	0.073	0.331	0.522	0.812	0.949	0.990	1.000
3 reads	0.089	0.383	0.616	0.887	0.977	0.996	1.000
4 reads	0.108	0.344	0.695	0.931	0.989	0.998	1.000
5 reads	0.129	0.556	0.760	0.958	0.994	0.999	1.000
10 reads	0.224	0.762	0.947	1.000	1.000	1.000	1.000
20 reads	0.397	0.939	0.997	1.000	1.000	1.000	1.000
30 reads	0.532	0.985	1.000	1.000	1.000	1.000	1.000

The proportion of CD19+ ASC's during acute dengue is well described [3,4]. The probability of sampling mB of 1 to 10 reads (size) was calculated using a fixed proportion of 56% of IgG+ ASC (≈ 5.6 % of CD19) (Figure 3B, main manuscript) and the following table:

**Sampling probability in Acute dengue**		
Size (reads)	mB (44%)	ASC (56%)
1	0.01543276	0.98456724
2	0.00023817	0.99976183
3	3.6756E-06	0.99999632
4	5.6725E-08	0.99999994
5	8.7542E-10	1
10	7.6637E-19	1

During the post-convalescent period, the ASC number in blood is very low. The majority of ASC's in peripheral blood are IgA plasmablast and plasma cells [5]. The proportion of peripheral blood B cells (CD19+) in humans is 9.2% ± 7.2–11.2 (IC range 25-75) [6]. The proportion of CD19+ B cells that express surface IgG (mB cells) is 9.7% (SD ±4.3) [7]. Intracellular IgG + ASC's constitute 0.04% (0.0-0.11) of total peripheral blood mononuclear cells [5]. Thus, we estimated that 4.3 % of IgG+ B cells are ASC's, and used such value to estimate the probability of sampling either a mB cell or an ASC of a given size (See figure 3C, main manuscript):

**Sampling probability in post-convalescence**		
Size (reads)	mB (96.7%)	ASC (4.3%)
1	0.64638845	0.35361155
2	0.41781803	0.58218197
3	0.27007275	0.72992725
4	0.17457191	0.82542809
5	0.11284127	0.88715873
10	0.01273315	0.98726685
15	0.00143682	0.99856318
20	0.00016213	0.99983787
30	2.0645E-06	0.99999794
40	2.6287E-08	0.99999997
50	3.3472E-10	1

References:

1. Halpern EF, Weinstein MC, Hunink MG, Gazelle GS. Representing both first- and second-order uncertainties by Monte Carlo simulation for groups of patients. Medical decision making : an international journal of the Society for Medical Decision Making. 2000;20(3):314-22.
2. Kelley DE, Perry RP. Transcriptional and posttranscriptional control of immunoglobulin mRNA production during B lymphocyte development. Nucleic acids research. 1986;14(13):5431-47.
3. Wrammert J, Onlamoon N, Akondy RS, Perng GC, Polsrila K, Chandele A et al. Rapid and massive virus-specific plasmablast responses during acute dengue virus infection in humans. Journal of virology. 2012;86(6):2911-8. doi:10.1128/JVI.06075-11.
4. Garcia-Bates TM, Cordeiro MT, Nascimento EJ, Smith AP, Soares de Melo KM, McBurney SP et al. Association between magnitude of the virus-specific plasmablast response and disease severity in dengue patients. J Immunol. 2013;190(1):80-7. doi:10.4049/jimmunol.1103350.
5. Mei HE, Yoshida T, Sime W, Hiepe F, Thiele K, Manz RA et al. Blood-borne human plasma cells in steady state are derived from mucosal immune responses. Blood. 2009;113(11):2461-9. doi:10.1182/blood-2008-04-153544.

6. Morbach H, Eichhorn EM, Liese JG, Girschick HJ. Reference values for B cell subpopulations from infancy to adulthood. Clinical and experimental immunology. 2010;162(2):271-9. doi:10.1111/j.1365-2249.2010.04206.x.
7. Fecteau JF, Cote G, Neron S. A new memory CD27-IgG+ B cell population in peripheral blood expressing VH genes with low frequency of somatic mutation. J Immunol. 2006;177(6):3728-36.
