# Supplementary material for: Lower IgG somatic hypermutation rates during acute dengue virus infection is compatible with a germinal center-independent B cell response
Source: Genome Med. 2016 Feb 25;8:23. doi: 10.1186/s13073-016-0276-1 (PMC4766701; doi:10.1186/s13073-016-0276-1)
Supplement: Additional file 2: — Clonotype and lineage clustering by ImmunediveRsity . Raw IgG HTS data was subjected to repertoire reconstruction and analysis using ImmunediveRsity. Briefly, ImmunediveRsity assigns IGHV gene to each sequence and clusters them according to IGH clonotypes, which are composed of reads with the same IGHV-IGHJ rearrangement and quasi-identical CDRH3 (≥97 % identity at the nucleotide level). Within a clonotype, one or more lineages can be defined based on SHM pattern (99.5 % identity). IGHV usage frequency can thus be expressed as the proportion of reads using a particular IGHV segment (relative transcription), regardless of clonotype origin, or can be expressed as the proportion of IGH clonotypes or lineages using a particular IGHV segment regarding the total amount of clonotypes or lineages, respectively. (PDF 35 kb) [file 13073_2016_276_MOESM2_ESM.pdf]

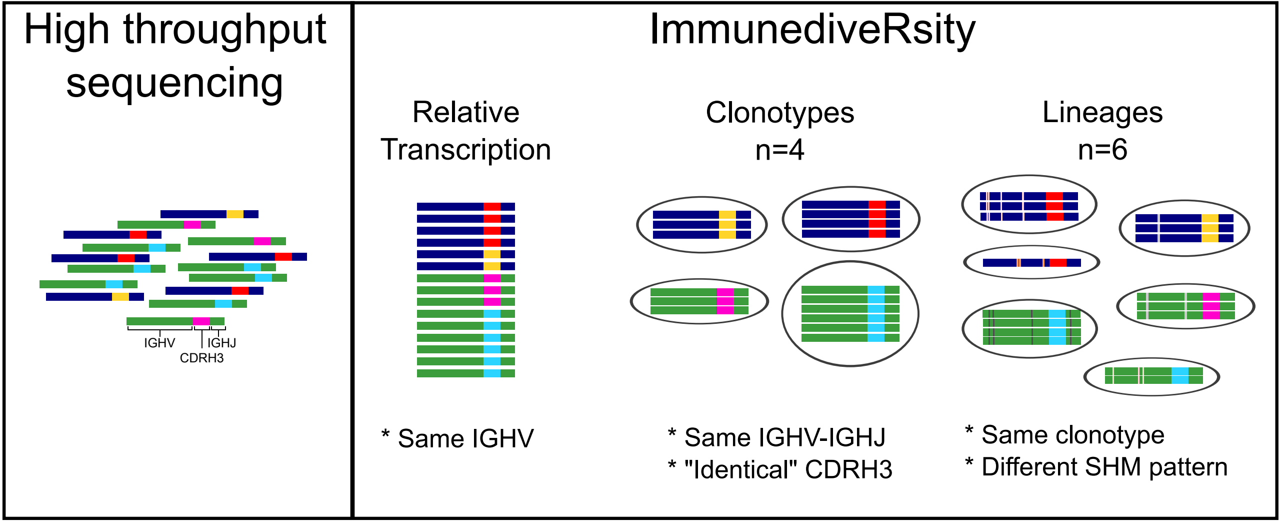
High throughput sequencing
IGHV
IGHJ
CDRH3
ImmunediveRsity
Relative Transcription
Clonotypes n=4
Lineages n=6
* Same IGHV
* Same IGHV-IGHJ
* "Identical" CDRH3
* Same clonotype
* Different SHM pattern
