# Supplementary figures and images for: Lower IgG somatic hypermutation rates during acute dengue virus infection is compatible with a germinal center-independent B cell response
Source: Genome Med. 2016 Feb 25;8:23. doi: 10.1186/s13073-016-0276-1 (PMC4766701; doi:10.1186/s13073-016-0276-1)

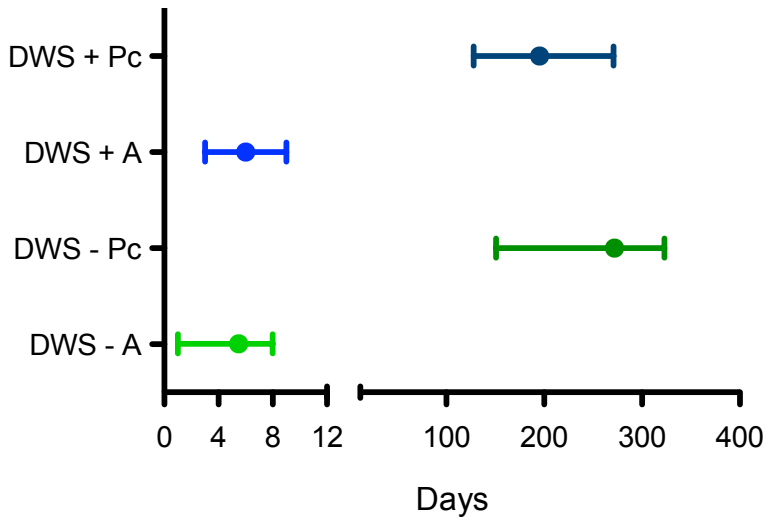

Supplement: Additional file 3: — Days after onset of clinical illness for blood sampling according to clinical status. DWS- A, green; DWS− Pc, dark green; DWS+ A, blue; DWS+ Pc, dark blue. (One-way ANOVA, p > 0.05, not significant). (PDF 16 kb) [file 13073_2016_276_MOESM3_ESM.pdf]

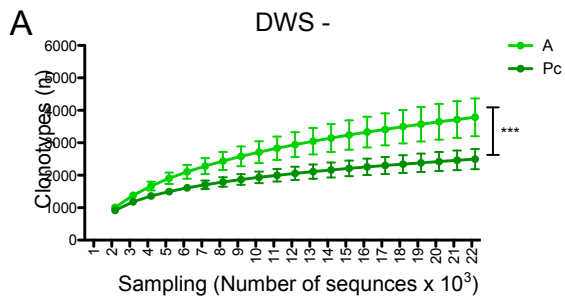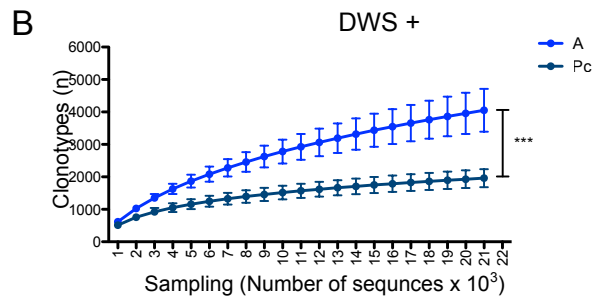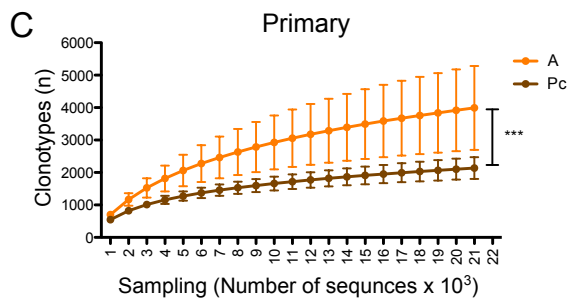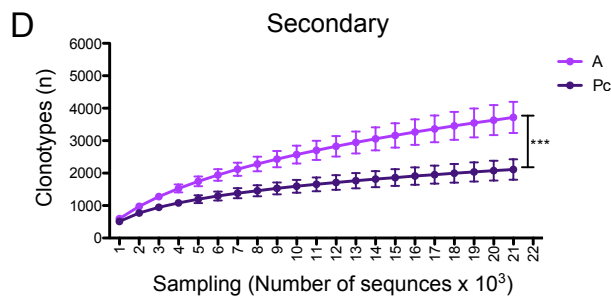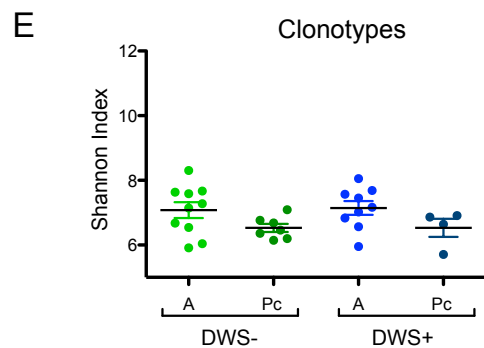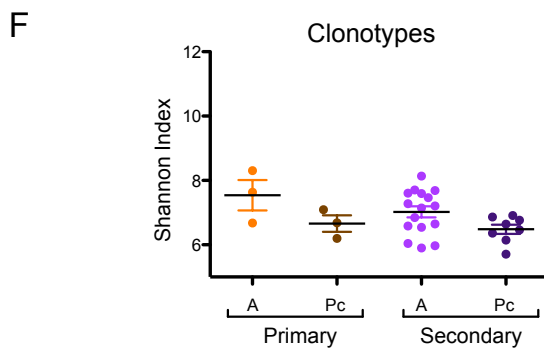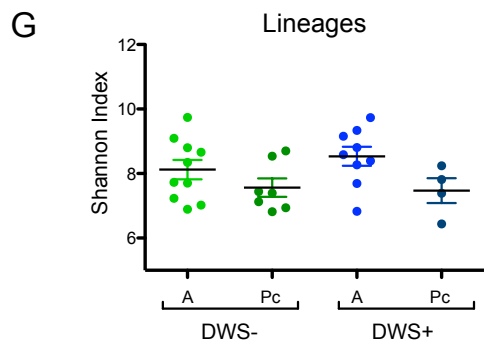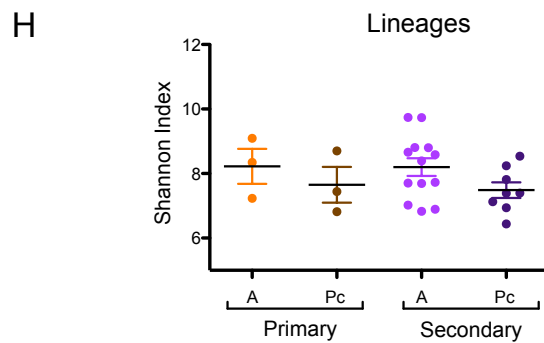

Supplement: Additional file 6: — Higher clonal diversity during acute phase than in the post-convalescent phase, regardless of clinical classification. Rarefaction analysis of acute vs. post-convalescent samples according to clinical status, (A) DWS− (A, green and Pc, dark green) and (B) DWS+ (A, blue and Pc, dark blue); and immune status, (C) primary (A, orange and Pc, brown) and (D) secondary infection (A, light purple and Pc, dark purple) (two-way ANOVA with Bonferroni correction, *p < 0.05; **p < 0.01). Shannon–Weaver entropy values for heavy chain clonotypes according to (E) clinical status and (F) immune status and for heavy chains lineages according to (G) clinical status and (H) immune status. (DWS− A, green; DWS− Pc, dark green; DWS+ A, blue; DWS+ Pc, dark blue; primary infection acute, orange; primary infection Pc, brown; secondary infection acute, light purple; secondary infection Pc, dark purple) (For E–H, one-way ANOVA with Bonferroni correction, *p < 0.05; **p < 0.01). (PDF 78 kb) [file 13073_2016_276_MOESM6_ESM.pdf]

A

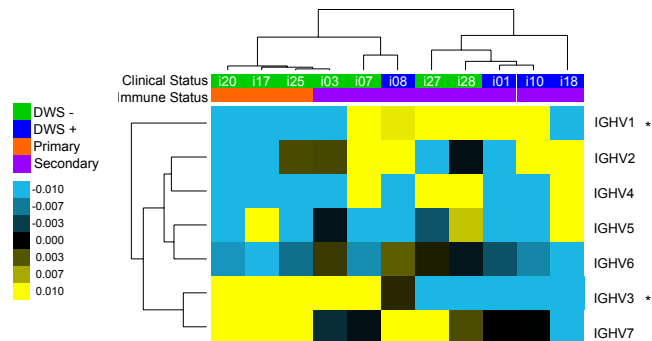

B

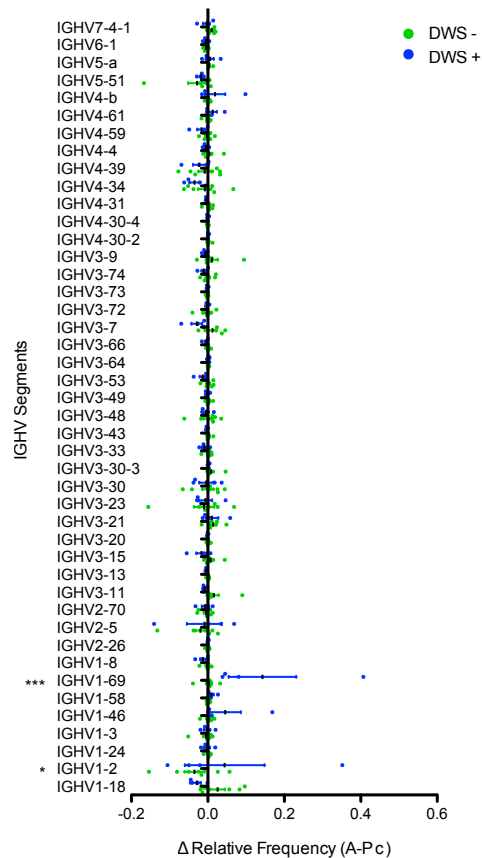

C

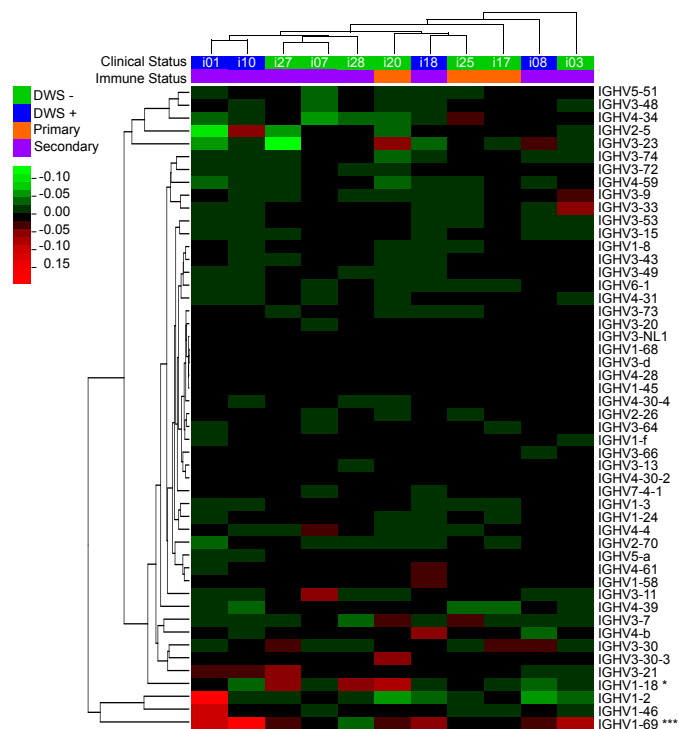

Supplement: Additional file 7: — Preferential IGHV transcription and usage during acute DENV infection. (A). Heat map of hierarchical clustering of the difference between acute minus post-convalescent read relative frequency (ΔA − Pc) per IGHV family. Over-transcription of IGHV family during acute phase is indicated in yellow tones, whereas blue tones indicate under-transcription. Upper rows classify patients according to clinical status (DWS−, green and DWS+, blue) and immune status (primary, orange and secondary, purple) (two-way ANOVA, Bonferroni correction for multiple testing, *p < 0.05). (B) Relative transcription difference between acute minus post-convalescent (ΔA − Pc) according to IGHV segment and clinical status. IGHV1-2 and IGHV1-69 are overexpressed in acute DWS+ (two-way ANOVA, Bonferroni correction for multiple testing, *p < 0.05). (C) Difference between the relative IGHV segment use per clonotypes (rows) in acute minus post-convalescent (ΔA − Pc) according to clinical status (columns: DWS−, green and DWS+, blue) and immune status (primary, orange and secondary, purple). Higher frequency of clonotypes using IGHV1-18 and IGHV1-69 in DWS+ is shown in red tones (two-way ANOVA, Bonferroni correction for multiple testing, *p < 0.05). (PDF 131 kb) [file 13073_2016_276_MOESM7_ESM.pdf]

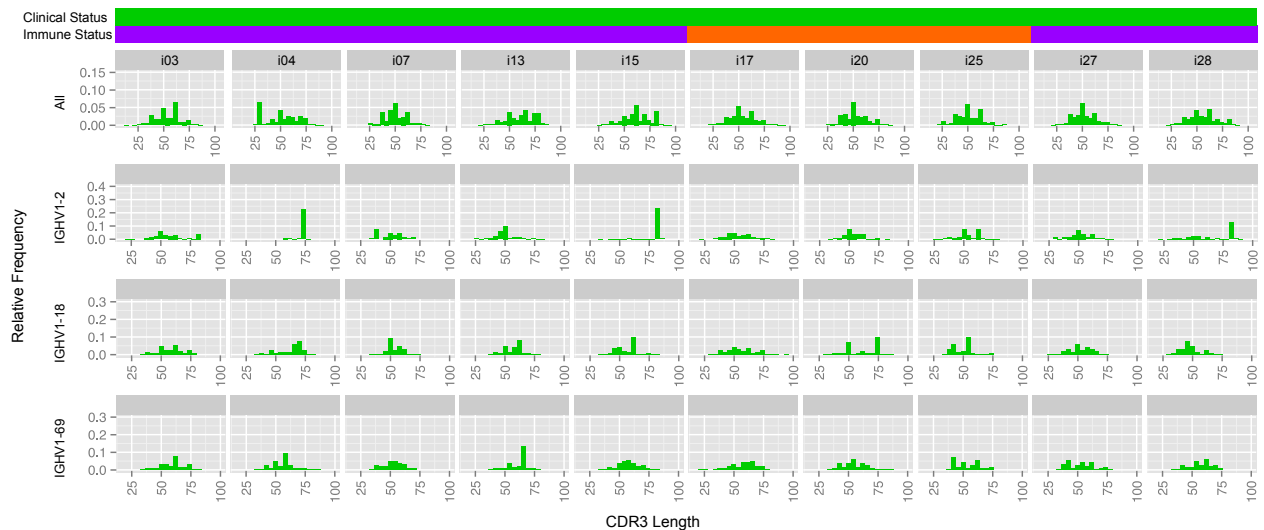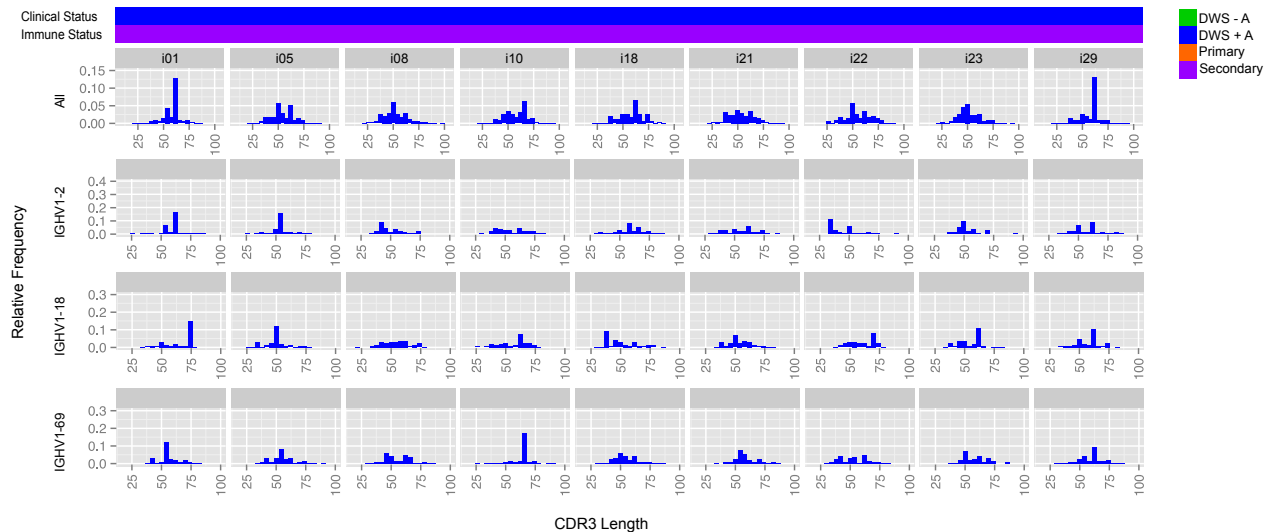

Supplement: Additional file 8: — Digital CDRH3 “spectratyping” of acute phase DENV infection. CDRH3 “spectratyping” of all segments and according to IGHV1-2, IGHV1-18, and IGHV1-69 segments based on the CDRH3 size of 1280 lineage subsample. Clinical and immune status is shown in upper bars. CDRH3 length (bp) is shown in x axis and lineage relative frequency is shown in y axis (color bars: DWS−, green and DWS+, blue. Upper lines: DWS−, green; DWS+, blue; primary, orange; secondary, purple). (PDF 619 kb) [file 13073_2016_276_MOESM8_ESM.pdf]

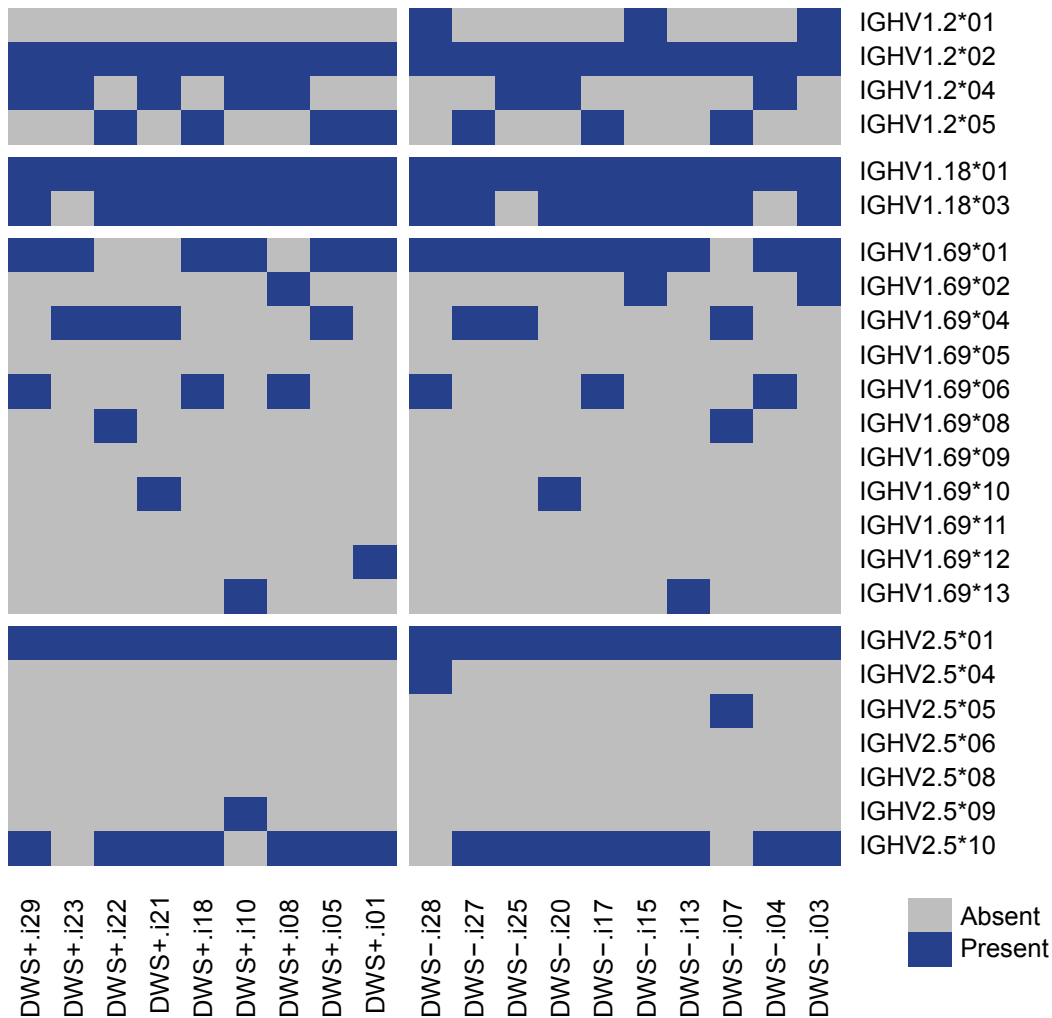

Supplement: Additional file 9: — IGHV allelic variation in patients with DENV infection. ImmunediveRsity output data in raw reads were used for allele calling. The matrix depicts each individual and the presence (dark blue) of absence (gray) of IGHV1-2, IGHV1-18, IGHV1-69, and IGHV2-5 alleles. (PDF 22 kb) [file 13073_2016_276_MOESM9_ESM.pdf]

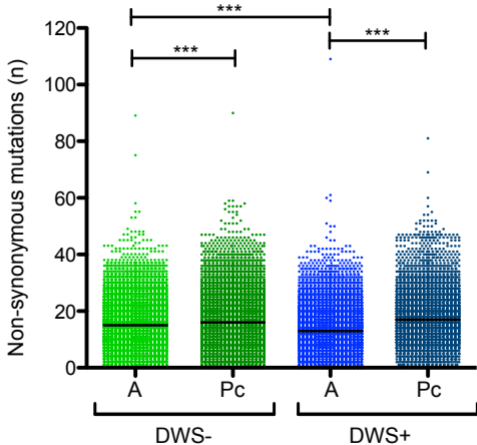

Supplement: Additional file 10: — Absolute number of non-synonymous mutations. Quantitative analysis of non-synonymous somatic hypermutation (DWS− A, green; DWS− Pc, dark green; DWS+ A, blue; DWS+ Pc, dark blue) (Kruskal–Wallis test for non-parametric data with Dunn’s multiple comparison test, *** p < 0.001). (PDF 1967 kb) [file 13073_2016_276_MOESM10_ESM.pdf]

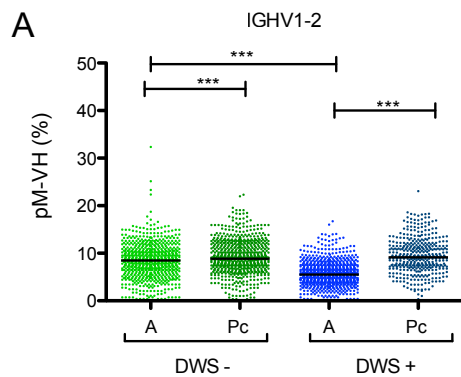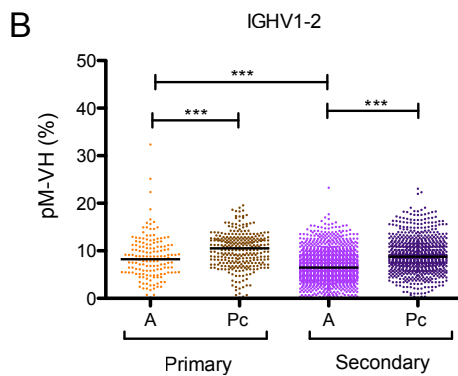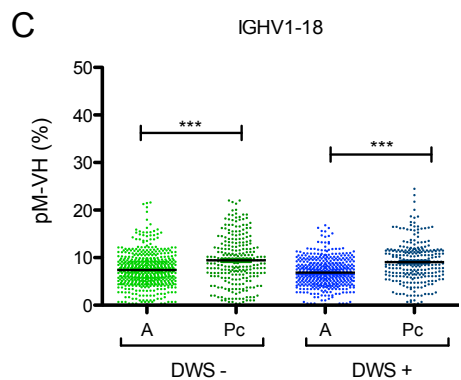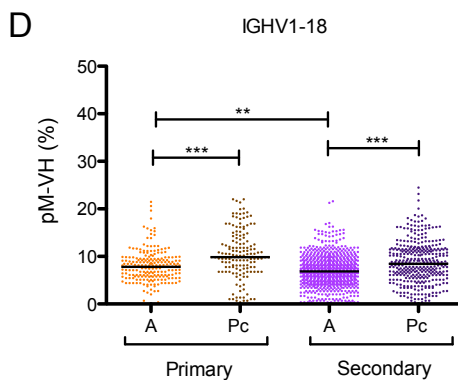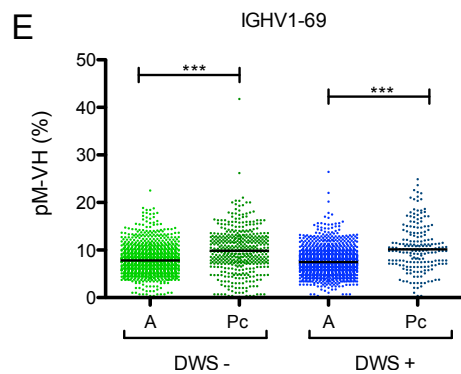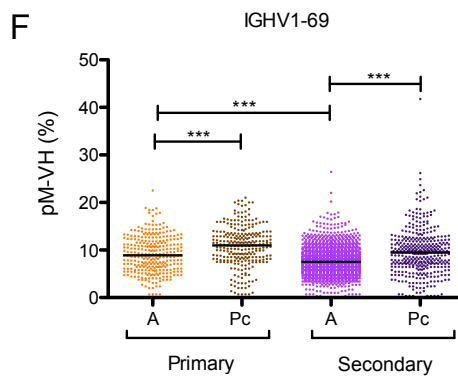

Supplement: Additional file 11: — Somatic hypermutation rates in selected IGHV segments according to clinical and immune status. SHM rates are shown according to clinical status (A, C, and E) and according to immune status (B, D, and F) measured as the proportion of mutations along the VH region [pM-VH (%)]. (A) SHM rates of IGHV1-2 regarding clinical status and (B) immune status. (C) SHM rates of IGHV1-18 regarding clinical status and (D) immune status. (E) SHM rates of IGHV1-69 regarding clinical status and (F) immune status. (DWS− A, green; DWS− Pc, dark green; DWS+ A, blue; DWS+ Pc, dark blue; primary infection acute, orange; primary infection Pc, brown; secondary infection acute, light purple; secondary infection Pc, dark purple) (Kruskal–Wallis test, Dunn’s correction for multiple testing, **p < 0.01, ***p < 0.001). (PDF 886 kb) [file 13073_2016_276_MOESM11_ESM.pdf]

A

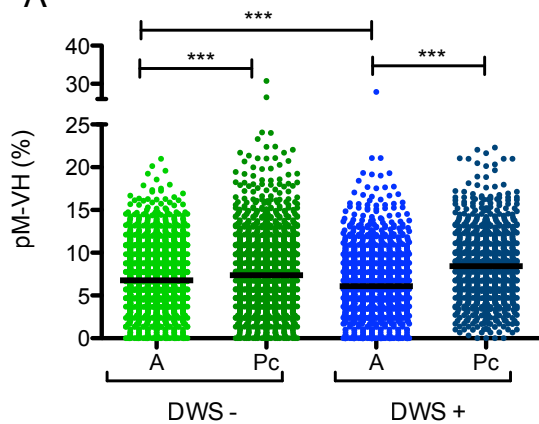

B

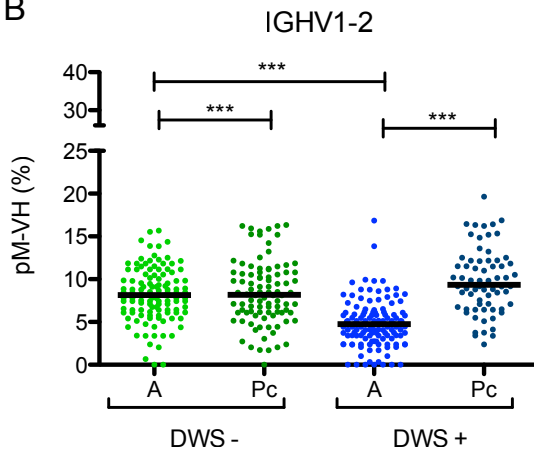

C

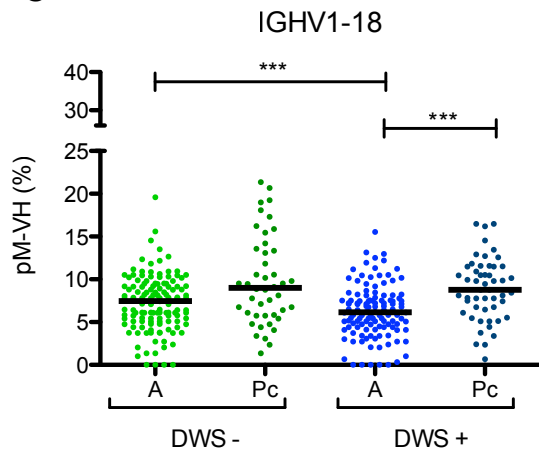

D

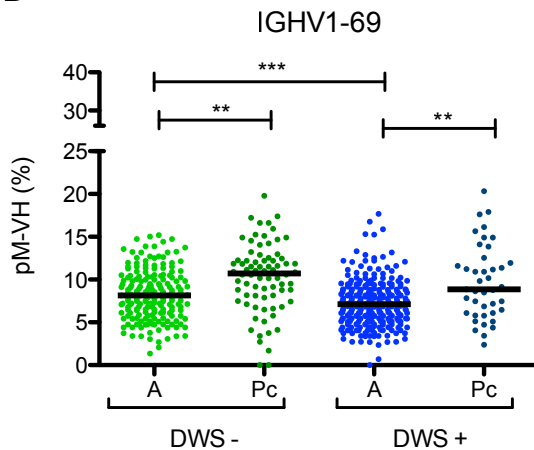

Supplement: Additional file 12: — SHM rates in global and selected IGHV segments of randomly subsampled clonotypes. Each dot represents the percentage of mutations in the largest lineage of 250 randomly sampled clonotypes per individual, classified according to clinical status. (A) Global mutation rates; (B) IGHV1-2; (C) IGHV1-18; and (D) IGHV1-69 (DWS− A, green; DWS− Pc, dark green; DWS+ A, blue; DWS+ Pc, dark blue) (Kruskal–Wallis test, Dunn’s correction for multiple testing, **p < 0.01, ***p < 0.001). (PDF 483 kb) [file 13073_2016_276_MOESM12_ESM.pdf]

A

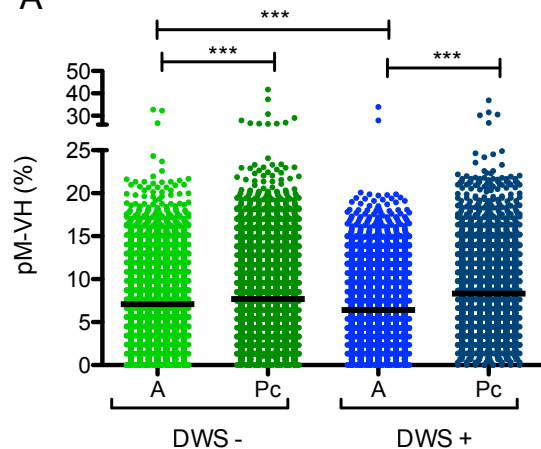

B

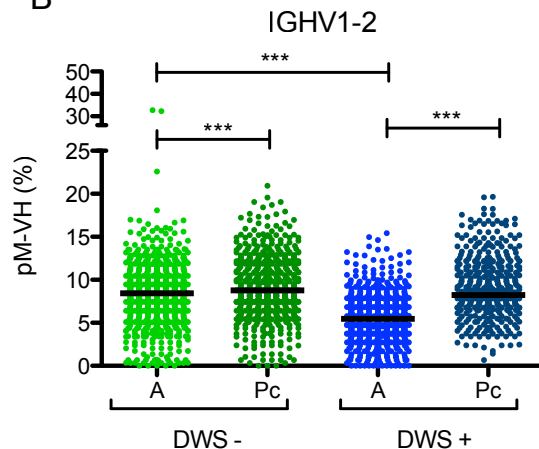

C

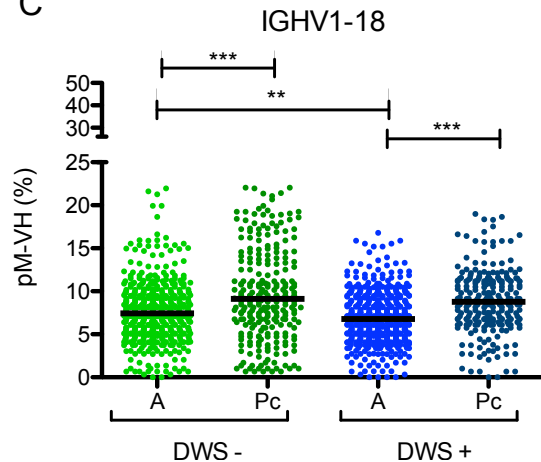

D

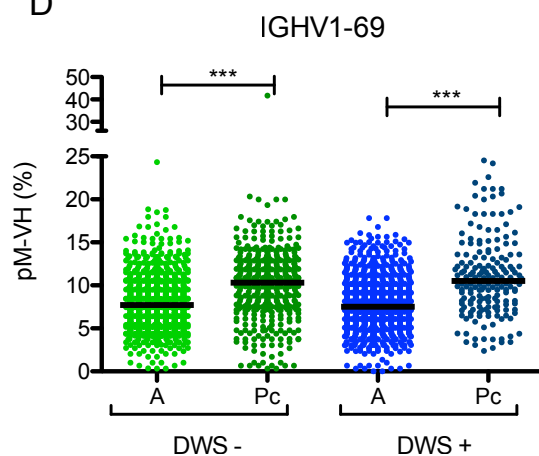

Supplement: Additional file 13: — SHM rates in global and selected IGHV segments in the ImmunediveRsity -reconstructed repertoire using a CDRH3 identity clustering threshold of 92 %. Each dot represents the percentage of mutations in randomly sampled lineages per individual, classified according to clinical status. (A) Global mutation rates; (B) IGHV1-2; (C) IGHV1-18; and (D) IGHV1-69 (DWS− A, green; DWS− Pc, dark green; DWS+ A, blue; DWS+ Pc, dark blue) (Kruskal–Wallis test, Dunn’s correction for multiple testing, **p < 0.01, ***p < 0.001). (PDF 2177 kb) [file 13073_2016_276_MOESM13_ESM.pdf]

■ DWS - A 
 ■ DWS - Pc 
 ■ DWS + A 
 ■ DWS + Pc 
 ■ Primary 
 ■ Secondary 
 ■ Healthy Control

\*\*\*

Clinical Status

Immune Status

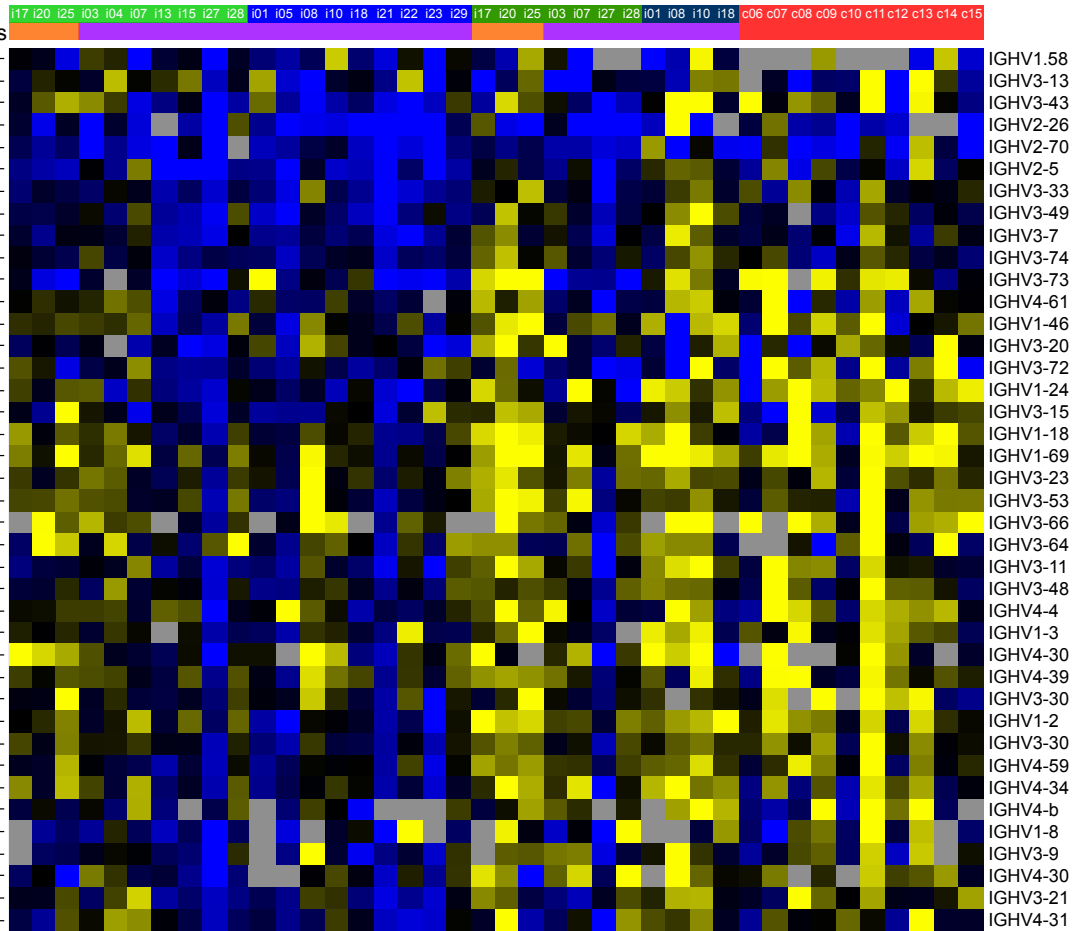

Supplement: Additional file 14: — Heat map representation of SHM according to IGHV segment ( rows ) and clinical status ( columns ). Acute DENV infection had significantly lower levels of SHM than post-convalescent and control samples (Mann–Whitney U test for non-parametric data, *** p < 0.001). (PDF 43 kb) [file 13073_2016_276_MOESM14_ESM.pdf]
